# Genetic structure of traditional cacao reveals four new genetic lineages in indigenous Amazonian sites in Peru

**DOI:** 10.1371/journal.pone.0351690

**Published:** 2026-07-06

**Authors:** Lambert A. Motilal, Martha S. Calderon, Danilo E. Bustamante, David Gopaulchan, Daniel Tineo, Fanny R. Márquez-Romero, Sphyros Lastra, Jorge R. Diaz-Valderrama, Juan Carlos Guerrero-Abad, Manuel Oliva, Pathmanathan Umaharan

**Affiliations:** 1 Cocoa Research Centre, The University of the West Indies, St. Augustine, Trinidad, Trinidad and Tobago; 2 Instituto de Investigación para el Desarrollo Sustentable de Ceja de Selva (INDES-CES), Universidad Nacional Toribio Rodríguez de Mendoza, Chachapoyas, Peru; 3 Instituto de Investigación en Ingeniería Ambiental (INAM), Facultad de Ingeniería Ambiental, Biosistemas y de la Energía (FIABE), Universidad Nacional Toribio Rodríguez de Mendoza, Chachapoyas, Peru; 4 School of Biosciences, University of Nottingham, Nottingham, United Kingdom; 5 Centro Experimental Yanayacu, Dirección de Servicios Estratégicos Agrarios (DSEA), Instituto Nacional de Innovación Agraria (INIA), Jaén, Peru; 6 Departamento académico de Ingeniería Agronómica Tropical, Universidad Nacional Intercultural de Quillabamba, Cusco, Peru; 7 Estación Experimental Agraria Perla del VRAEM, Dirección de Recursos Genéticos y Biotecnología, Instituto Nacional de Innovación Agraria (INIA), Cusco, Perú; 8 Instituto de Investigación, Innovación y Desarrollo para el Sector Agrario y Agroindustrial (IIDAA), Facultad de Ingeniería y Ciencias Agrarias (FICA), Universidad Nacional Toribio Rodríguez de Mendoza de Amazonas, Chachapoyas, Peru; Institute for Biological Research, University of Belgrade, SERBIA

## Abstract

Cacao genetic resources in Peru are largely uncharacterized. Knowledge of their genetic structure is needed for their conservation and use. Indigenous on-farm cacao trees (n = 390) from the Departments of Amazonas (n = 130), Ayacucho (n = 76), Cajamarca (n = 1), Cusco (n = 110), Madre de Dios (n = 10), Piura (n = 59), San Martín (n = 3) and Ucayali (n = 1) in Peru were fingerprinted with 192 single nucleotide polymorphic markers. Identity, group differentiation, phylogenetic, multivariate and ancestry analyses were conducted. Four new populations were identified guided by phylogenetic, accepted ancestral backgrounds of reference samples and least admixture among samples. The cacao from these eight Departments were variably mixed containing both pure members of new populations and admixed samples with these new populations and Amelonado, Contamana, Criollo, Curaray, Guiana, Iquitos, Marañon, Nacional, Nanay and Púrus. The findings of this study suggest that while the cacao germplasm is genetically related across different departments, each region harbors its own distinct genetic composition. The Clade IV (Chuncho 2) population was associated with the Contamana cluster and Clade I (Chuncho 1) was associated with Purus cluster. Clades II (Awajun) and III (Porcelana) were associated with the Nacional cluster. The ancestry of the economically desired CCN 51 cultivar was revealed to be better assigned as 15% Amelonado, 13% Criollo, 25% Iquitos and 45% Awajun. The results of this study will improve the understanding of the genetic landscape in Peru, enhance genebank collections in Peru and enable the differentiation of the fine flavour industry in Peru. The new groups of cacao identified in this study will help understand the genetic structuring of cacao and represent a valuable new resource to search for valuable traits for breeding and commercialization programmes in the fine flavour cacao industry in Peru.

## Introduction

Cacao (*Theobroma cacao* L.), an outcrossing understorey tree species native to the Amazon basin, holds significant economic value due to its beans [1]. These cocoa beans serve as the primary raw material for the multibillion dollar chocolate industry and are also used in various food and cosmetic products, making cocoa beans a vital agricultural commodity [2]. Cacao cultivation remains a crucial income source for millions of small-scale farmers in tropical areas, playing a pivotal role in global trade and the economies of producer nations [3]. Additionally, *T. cacao* possesses extensive genetic diversity, attracting scientific research aimed at improving yield, disease resilience, and bean quality through targeted breeding programs [2].

Peru ranks as the eighth-largest cocoa producer and tenth-largest exporter of cocoa beans in the world [4]. Globally recognized for its premium fine-flavor cocoa [5], Peru is also the second-largest producer of organic cocoa in the world [6]. As of 2024, cocoa cultivation supports 83,294 Peruvian farming families, generating 156,625 tons annually with a yield of 0.826 t ha ⁻ ¹ [7]. The San Martín region dominates production with 63,033 tons (40.2% of national output), followed by Junín (35,297 tons, 22.5%), Huánuco (17,132 tons, 10.9%), Cusco (10,909 tons, 7.0%), Ucayali (8,050 tons, 5.1%), Pasco (5,128 tons, 3.3%), and Amazonas (4,847 tons, 3.1%) [7]. Collectively, these seven regions account for 92.2% of Peru’s total production.

A seminal classification of the molecular genetic diversity of cacao established ten genetic groups (Amelonado, Contamana, Criollo, Curaray, Guiana, Iquitos, Marañon, Nacional, Nanay, and Purús) [8]. This framework has since been supplemented by findings of putative novel genetic groups in several countries. For instance, the genetic groups Apaporis, Caqueta, Pangui, Napo and Tiwinza were found in Ecuador and Colombia [9]; Cacao Nacional Boliviano in Bolivia [10]; and Piura Porcelana and Chuncho in Peru [11]. However, the status of these populations remains tentative due to limitations in the respective studies inclusive of suboptimal clustering, missing genetic clusters, and few representatives in a genetic group [12]. The proposed groups were probably at the expense of established populations and further modelling and sampling are required to firmly establish whether these are new genetic clusters, subgroups of existing populations or sister clades of related germplasm [12]. This complexity is illustrated by the analysis of northern Peruvian cacao, which identified three genetic clusters (PhyloA, PhyloB and PhyloC) [12]. Although phylogenetic modelling supported their distinctness, population modelling separated these clusters at the expense of the established clusters of Motamayor et al. [8]. Bustamante et al. [12] therefore interpreted the new genetic clusters as hybrid clades with ancestry traceable to the established groups defined by Motamayor et al. [8].

Consequently, although studies on the genetic diversity of cacao (particularly fine-aroma varieties) in Peru are scarce, additional efforts in DNA fingerprinting, supported by robust modelling and expanded sampling, are critical for optimally demarcating genetic groups. Therefore, this study aimed to determine the genetic uniqueness, diversity, and ancestry of on-farm indigenous Amazonian cacao trees in Peru using SNP genotyping. We also investigated whether these trees harbour novel genetic clusters. The resulting findings are expected to significantly enhance our understanding of the genetic diversity of wild and semi-wild primary cacao germplasm in Peru which can be leveraged to improve the cacao industry in Peru.

## Materials and methods

### Plant material and sampling

**Fig 1:**
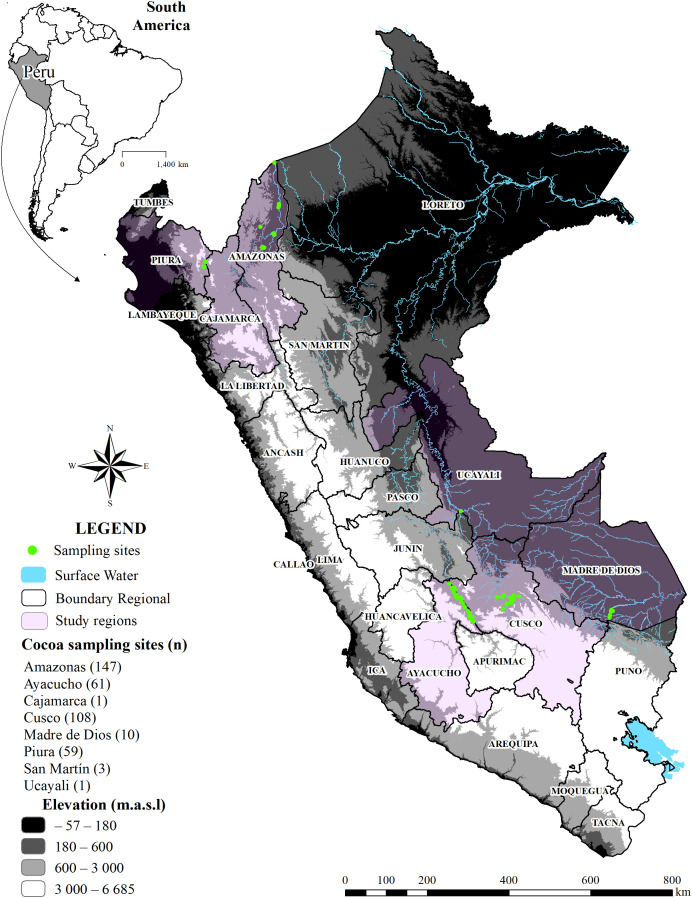
Sampling locations and sample sizes for cacao in Peru. Amazonas (n = 130), Ayacucho (n = 76), Cajamarca (n = 1), Cusco (n = 110), Madre de Dios (n = 10), Piura (n = 59), San Martín (n = 3), and Ucayali (n = 1). The political-administrative boundary layers were obtained from the Spatial Data Infrastructure of the National Institute of Statistics and Informatics of Peru (INEI), which are freely accessible (https://ide.inei.gob.pe/#capas). These layers correspond to the political-administrative boundaries of Peru and were used only as spatial reference layers for the elaboration of the map. The hydrographic layer, including rivers and streams, was obtained from the dataset “Hidrografía de Perú” on the Humanitarian Data Exchange (HDX) (https://data.humdata.org/dataset/hidrografia-de-peru). According to the dataset description, this information corresponds to basic cartographic data for Peru, generalized from the National Map at 1:100,000 scale. The dataset is made available through HDX under a Creative Commons Attribution for Intergovernmental Organisations (CC BY-IGO) license.

Indigenous on-farm Amazonian cacao trees (n = 390) were sampled from Peru in the Amazonas (Bagua, n = 16; Condorcanqui, n = 32; Utcubamba, n = 82), Ayacucho (Huanta, n = 3; La Mar, n = 73), Cajamarca (Jaén, n = 1), Cusco (La Convención, n = 110), Madre de Dios (Inambari, n = 10), Piura (Huancabamba, n = 59), San Martin (Mariscal Caceres, n = 3) and Ucayali (Atalaya, n = 1) departments (Fig 1 and S1 Table). Plant material was collated over two collection expeditions with 111 samples sourced from the first collection [12] and 279 newly sampled material in the second collection.

Trees that were about 50 years of age were selected based on traditional knowledge of farmers. These trees mainly represent the traditional lineages of unimproved cacao varieties and may be considered as wild and semi-wild primary germplasm. Samples from the Amazonas and Cajamarca departments were included as earlier work indicated that they formed three distinct phylogenetic groups [12]. Recent collection included 23 newly sampled trees from the Condorcanqui province (northern Amazonas department). A permit for scientific research on wild flora (RDG N° D000319-2020-MINAGRI-SERFOR-DGGSPFFS, with authorisation code N° AUT-IFL-2020–051) was provided by Servicio Nacional Forestaly de Fauna Silvestre (SERFOR).

Population references of the 10 genetic clusters of Motamayor et al*.* [8] originated from the cacao SNP fingerprinting dataset, inclusive of accessions from International Cocoa Genebank Trinidad (ICGT), managed by the Cocoa Research Centre (CRC) were used. These reference samples have been fingerprinted as part of the CRC germplasm molecular management strategy and have been reliably allocated to their home populations by ancestry and phylogenetic studies. The Amelonado, Contamana, Criollo, Curaray, Guiana, Iquitos, Marañon, Nacional, Nanay and Purús clusters were represented by 70, 15, 36, 18, 62, 56, 162, 104, 83, and 5 individuals, respectively. These individuals had exclusive (>97%) membership to the home populations and with less than 2.5% membership to any other genetic cluster of Motamayor et al. [8]. These representative population individuals were used as training samples to simulate 2000 samples in each of the 10 genetic clusters using the software WHICHLOCI [13] and 186 polymorphic SNPs with the least missing data in the representative population individuals. This allowed the generation of equivalently sized genetic clusters in which missing data was absent. The actual accessions were not used in the data analyses and instead the simulated genetic clusters were used as the reference genetic clusters corresponding to the 10 groups in Motamayor et al. [8]. Selected accessions in the ICGT with known Amelonado-Criollo (28 accessions) or Amelonado-Nacional (28 accessions) admixture were included as they are known to have fine flavour attributes and are commonly found admixed individuals and which may form their own phylogenetic clades. The commercial cultivar CCN 51 was also included as it is an increasingly popular planting material and is a known admixed accession [14]. The Amelonado-Criollo and the Amelonado-Nacional reference samples had variable ancestral contribution from their respective parental clusters and with less than 5% ancestry in any other genetic cluster described in Motamayor et al. [8]. These 57 accessions form the ICGT reference accessions in this study. The following methodology are summarized in S1 Fig.

### DNA fingerprinting

The cacao fingerprinting panel of 192 SNPS developed at CRC [15,16] and previously used in Bustamante et al*.* [12] was screened across the 390 samples from Peru. The BioArk leaf collection kits from LGC Biosearch Technologies were used and the plates were shipped to LGC Genomics, United Kingdom for DNA extraction and SNP genotyping using their proprietary Kompetitive Allele Specific PCR (KASP™) assays [17]. The KASP™ process uses two different allele-specific competing forward primers with unique tail sequences and one common reverse primer. Fluorophores are provided as quenched sequences complementary to the tails of the anticipated amplified sequences. After initial amplification of the competing primers, the reverse primer generates a complementary sequence to which the fluorophores bind thereby becoming unquenched and releasing the coloured signal for that SNP allele. Data on biallelic SNPs are provided by the KASP™ process

### Identity analysis

The cacao fingerprinting dataset (6831 unique profiles from over 27,000 samples in 32 countries) maintained by the Cocoa Research Centre (CRC) of The University of the West Indies, St. Augustine Campus, Trinidad and Tobago was used for identity analysis. The 390 sampled trees from Peru (S2 Table) were compared to each other and to the CRC cacao fingerprinting dataset using the software Cervus v3.0 [18]. Three samples (DBMC041, DBMC052 and DBMC_R7) had the most missing data at 14.1%, 27.6% and 60.9% respectively. Since only 75 SNPs were present in DBMC_R7, a minimum match number of 60 SNPs with a fuzzy mismatch at 5 SNPs was implemented in Cervus v3.0 [18]. Match declarations based on the number of matches, mismatches and the presence of missing data is presented in Table 1. Samples within Match Groups are highly related or identical to each other and were considered duplicates. Samples between Match Groups may be similar to each other but were not considered as duplicates. The conservative combined non-exclusion probability of identity if individuals are full siblings (PID_sib_) was reported [19] as employed by Zhang et al. [20] in cacao.

**Table 1:** Classification criteria for SNP-based matching categories. Thresholds for determining genetic similarity between sample pairs based on exact matches, differences, and missing data across a 192-SNP panel.

Match category	# SNPs exactly matched in panel of 192 SNPs	# SNPs different	Missing data within a sample pair
Identical	180-192	0	absent
	≥ 188	0-1	absent or present
Very similar	186-187	0-1	absent
	≥ 186	2-3	absent or present
Similar	184-185	0-3	absent or present
Fairly similar	175-184	1-3	absent or present
	170-175	0	absent
Dissimilar	186-188	≥ 4	absent or present
	<186	≥ 4	absent or present

### Data reduction

The three samples with most missing data were retained for ancestry analysis but were excluded from all other analyses. One SNP (TcSNP0383) was monomorphic and was removed from the analyses. Twelve SNPs (TcSNP0198, TcSNP0230, TcSNP0259, TcSNP0280, TcSNP0456, TcSNP0577, TcSNP0644, TcSNP0701, TcSNP1038, TcSNP1229, TcSNP1230 and TcSNP1408) had at least 20% missing data in at least one of the sample clusters from Peru and were removed from the datasets except for ancestry analysis. Missing data in the Peru dataset (387 samples/174 SNPs) ranged from 0–5.2% with 0.54 ± 0.03 mean percentage missing data. Simulated samples have no missing data and the 57 ICGT reference accessions had a mean of 0.02 ± 0.01 percentage missing data across the 174 SNP markers. In ancestry analysis, the maximal number of SNPs was

### Collection group differentiation

Departmental groups with less than five samples for the 174 SNP markers were removed. The dataset was further reduced by selecting one sample to represent each MatchGroup unless the samples were from different departments in which case, each department retained one member of the appropriate MatchGroup. The 10 genetic clusters of Motamayor et al*.* [8] were each represented by 100 simulated samples. The hybrid Amelonado groups with Nacional (28 samples) and Criollo (28 samples) were retained. The group differentiation D_est_ test [21,22] was implemented in GenAlEx v6.502 [23,24] with 999 permutations and 999 bootstraps. The D_est_ analyses were based on (a) the Department from which the samples were collected and (b) using previously identified phylogenetic groups of Motamayor et al. [8] and Bustamante et al. [12].

### Principal coordinate analysis

Principal coordinate analyses (PCoAs) were conducted in GenAlEx v6.502 [23,24] on all the unique individuals per Department and on the D_est_ matrix of the group differentiation test. A linear genetic distance was used to generate the distance matrix for the dataset of all individuals and implemented the distance-standardised option. The PCoA analyses were based on (a) the Department from which the samples were collected and (b) using previously identified phylogenetic groups of Motamayor et al. [8] and Bustamante et al. [12].

### Phylogeny

The multilocus profiles of the 387 samples from Peru (inclusive of duplicates), 100 simulated samples in each of the 10 genetic clusters of Motamayor et al*.* [8], CCN 51, 28 Amelonado-Nacional accessions and 28 Amelonado-Criollo accessions from the reduced set of 174 SNP markers formed the phylogeny dataset. GenAlEx v6.502 [23,24] was used to create the infile for DARwin v6 [25,26]. In the program, minimal proportion of valid data per unit pair was set at 80% using the default pairwise allele deletion to create simple matching dissimilarity index matrices with 1,000 bootstraps. The default weighted Neighbor Joining algorithm with 1,000 bootstrap replicates was used to generate the cladogram. Bootstrap values of at least 80% were displayed on a tree rooted at the Criollo clade.

### Ancestry

The complete set of 390 samples from Peru in conjunction with that of 2000 simulated samples in each of the 10 genetic clusters of Motamayor et al. [8] at 186 SNP loci was used in GenAlEx v6.502 [23,24] to create the infile for STRUCTURE v2.3.4 [27]. The command line option was employed with *a priori* settings using 200,000 burnins and 300,000 Markov chain Monte Carlo (MCMC) samplings. An admixture model with independent allele frequencies was employed to generate 20 iterations at 5–20 populations (K) inclusive. The *ad hoc* method of Evanno et al*.* [28] and the 10 clusters of Motamayor et al*.* [8] were used to guide the selection of the optimal number of populations. This was considered as Phase I ancestry analysis.

In Phase II ancestry analysis, checks were conducted on defined clusters of the wild samples based on the phylogenetic analysis. Input details were as above, except that there were 100 simulated samples in the reference populations. Each phylogenetic clade containing the Peru samples was used as a population in separate ancestry projects. The number of clusters was set at K = 11 and 10 iterations were run. Runs were assessed for correct allocation of the 10 reference populations of Motamayor et al. [8]. Pure members (>90% in new cluster and <5% in any of the 10 reference populations) were identified for each clade and were used in

Phase |IV anacestry analysis. In addition, two ancestry checks were conducted by using the 10 simulated population groups, CCN 51 and either the 28 Amelonado-Criollo individuals or the 28 Amelonado-Nacional individuals as a putative group. Ten iterations at K = 11with run settings as described earlier were undertaken.

In Phase III, a reanalysis in STRUCTURE v2.3.4 [27] of the 10 reference populations together with the collated pure members from each clade and 11 reference samples (CCN 51, five Amelonado-Criollo samples, five Amelonado-Nacional samples) was then undertaken to confirm the optimal number of populations. Ten to fifteen iterations at 1–19 clusters were run with settings as described earlier.

In Phase IV ancestry analyses, all of the Peru samples were reassessed in STRUCTURE v2.3.4 [27,28] using the optimal number of populations and the chosen new pure cluster(s) from the recently collected Peru samples. Additional ancestry runs were performed as needed to confirm population assignment and ancestry estimates.

Ancestry runs were assessed for correct separation of clusters, cluster allocation and assignment of the 11 reference samples. The iteration with the most positive lnP(D) was used to represent the ancestral estimates in Phases I – III inclusive. Final ancestry estimates were obtained in StructureSelector [29] with CLUMPAK [30] running the LargeKGreedy Algorithm and 2000 random input orders repeats.

## Results

### Identity analysis

Twenty-five match groups (Table 2) were obtained among the samples from Peru all of which originated from the second collection (n = 279). Duplicate samples were mainly found within departments (22 groups) than among departments (3 cases) and were present in increasing frequency in the Piura (6 groups), Ayacucho (9 groups) and Cusco (13 groups) departments. Most of the match groups (23) were composed of two members except for Match Group 3 with three members and Match Group 4 with eight members from Cusco. Comparison to the 6831 unique multilocus profiles resulted in only one match. The match pair was CCA015 (Condorcanqui, Amazonas) to CCN 51 with a PID_sib_ of 8.19 × 10^-34^ matches at 182 SNPs and 0 mismatches out of a possible 185–187 loci. A PID_sib_ of 7.082 × 10^-34^ was obtained in the entire dataset of reference and Peru samples. The sample DBMC007 could be included in Match Group 4 with 177 matched SNPs and only one mismatched SNP out of 178 typed loci. DBMC_A20 with 178 SNPs was matched to Match Group 14 at 175 SNPs and mismatched at 2 SNPs and DBMC_P12 was matched to Match Group 17 at 179 of 185 SNPs and mismatched at 4 SNP loci. There were four cases of closely related but likely dissimilar samples. Match Group 14 and Match Group 15 could be fairly similar to each other being matched at 172–179 SNPs and with 3–5 SNP mismatches. Samples CHMM09 and CHMM10 both from La Mar in Ayacucho was closely related but dissimilar being matched at 183 of 188 loci and mismatched at four SNP loci. Samples DBMC_A16 and DBMC_A17 both from Huancabamba, Piura were matched at 180 and mismatched at 5 SNPs from a total of 185 typed loci. Samples DBMC_P16 and DBMC_P20 were also both from Huancabamba, Piura and were matched at 181 and mismatched at 4 SNPs from a total of 185 typed loci.

**Table 2:** Match groups with duplicated genotypes of cacao sampled in Peru. ^a^bracketed member may be in group but has less typed loci. ^b^% match does not include the bracketed group member in the members column.

Match group (MG)	Number of members	Departments	Members^a^	Minimum % match (number markers matched of number typed)^b^	Number of differing SNPs
MG01	2	Ayacucho, Cusco	CER03, VRAE99	100% (189 of 189)	0
MG02	2	Ayacucho, Cusco	CHMM01_P1, CHMM01_P2	(186 of 189)	1
MG03	3	Ayacucho, Cusco	CHMM05, CHMM08, CPV01_P1	(187–188 of 188–189)	0
MG04	8 or 9	Cusco	CHMM07, DBMC014, DBMC030, DBMC103, DBMC104, DBMC106, DBMC107, DBMC108, (DBMC007)	(186–188 of 186–189)	0
MG05	2	Cusco	CHMM12, CPV82	(185 of 187–188)	1
MG06	2	Ayacucho	CHMM13, CHMM14	(187 of 189)	2
MG07	2	Cusco	CPV01_P3, CPV01_P4	(187 of 187–189)	0
MG08	2	Ayacucho	CPV03, CPV05	(187 of 188–189)	1
MG09	2	Ayacucho	CPV08, CPV26	(185 of 187)	0
MG10	2	Ayacucho	CPV25, CPV34	(187 of 187–188)	0
MG11	2	Ayacucho	CPV27, CPV29	(187 of 187–188)	0
MG12	2	Cusco	CPV78, CPV79	(186 of 187–189)	1
MG13	2	Piura	DBMC_A18, DBMC_A20	(175 of 178–185)	3
MG14	2 or 3	Piura	DBMC_P15, DBMC_P19, (DBMC_A20)	(184 of 184)	0
MG15	3	Piura	DBMC_A19, DBMC_P6, DBMC_P8	(178–180 of 182–185)	1-3
MG16	2	Piura	DBMC_P10, DBMC_P11	(184 of 185)	0
MG17	2 or 3	Piura	DBMC_P13, DBMC_P18, (DBMC_P12)	(183 of 184–185)	0
MG18	2	Piura	DBMC_R10, DBMC_R8	(183 of 184–185)	0
MG19	2	Cusco	DBMC004, DBMC102	(186 of 186–187)	0
MG20	2	Cusco	DBMC027, DBMC037	(188 of 188–189)	0
MG21	2	Cusco	DBMC041, DBMC049	(165 of 165–189)	0
MG22	2	Cusco	DBMC081, DBMC082	(186 of 186–189)	0
MG23	2	Cusco	DBMC085, DBMC086	(187 of 188)	0
MG24	2	Cusco	DBMC097, DBMC098	(185 of 187–188)	1
MG25	2	Ayacucho	VRAE30, VRAE86	(187 of 188)	0

### Group differentiation

**Fig 2:**
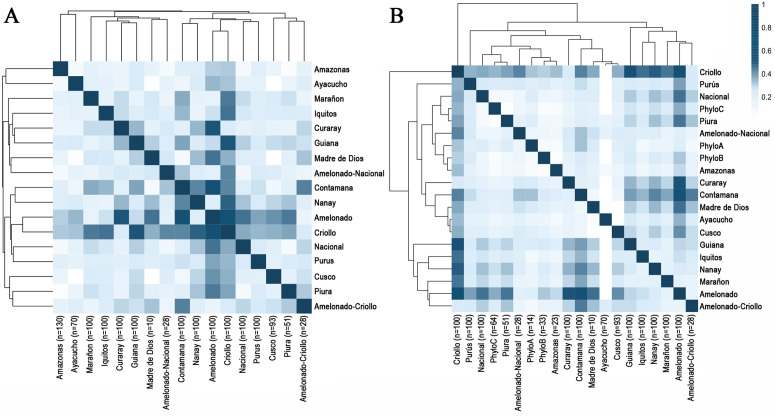
Group differentiation D_est_ statistic among based on (A) Departments from which samples were collected and (B) prior phylogenetic clusters. Heatmap are showing D_est_ values based on 999 permutations of populations (n = number of individuals). PhyloA, B and C are the clades identified in Bustamante et al. [12].

The 17 groups based on departmental groupings were all significantly different from each other (P = 0.001–0.015; Fig 2A) with maximal D_est_ value of 0.610 reported in the Amelonado-Criollo pair group. Among the five departments, the closest was Ayacucho-Cusco (D_est_ = 0.010) and the most distant was Madre de Dios-Piura (D_est_ = 0.180).

All 20 putative genetic clusters were devoid of private alleles but were significantly different from each other (P = 0.001–0.015; Fig 2B). Seven pairwise groups were the most differentiated (D_est_ ≥ 0.450) and involved eight clusters, seven of which were among the established clusters of Motamayor et al. [8] and the other was the set of newly collected samples from Madre de Dios. These seven pairwise groups always involved the Amelonado and Criollo clusters (Amelonado/Contamana, Amelonado/Curaray, Amelonado/Criollo, Amelonado/Madre de Dios, Guiana/Criollo, Iquitos/Criollo, Nanay/Criollo). The PhyloA-Madre de Dios pair was the most separated pair (D_est_ = 0.232) among the newly collected samples from Peru.

The closest clusters of Motamayor et al. [8] were Iquitos-Nanay, Guiana-Marañon, Amelonado-Marañon, Iquitos-Marañon, Curaray-Nacional, and Marañon -Nanay with D_est_ values of 0.141, 0.151, 0.152, 0.161, 0.165 and 0.167 respectively. Twenty-six pairwise groups were the least differentiated (D_est_ ≤ 0.109) and all included the eight putative clusters (PhyloA, PhyloB, PhyloC, Amazonas, Ayacucho, Cusco, Madre de Dios, Piura) recently collected in Peru, as well as, these clusters with the Contamana, Iquitos, Nacional, Amelonado-Criollo and Amelonado-Nacional clusters (Fig 2). The number of close groupings varied among the putative Peru clusters. Cusco and Madre de Dios had the lowest number (3) of close pair groups (Cusco vs. Contamana, Ayacucho, Madre de Dios; Madre de Dios vs. Contamana, Ayacucho, Cusco). Amazonas had the highest number (8) of close pair groups (Amazonas vs. Ayacucho, Iquitos, PhyloA, PhyloB, PhyloC, Piura, Nacional, Amelonado-Nacional).

The closest pairs were Ayacucho-Cusco, Madre de Dios-Cusco, PhyloC-Piura, PhyloB-Amazonas, Ayacucho-Madre de Dios, PhyloB-PhyloC, PhyloC-Amazonas, PhyloA-Amazonas and PhyloA-PhyloB with D_est_ values of 0.010, 0.015, 0.020, 0.026, 0.030, 0.040, 0.042, 0.043 and 0.053 respectively. The Ayacucho-Cusco and Madre de Dios-Cusco pair groups were the least differentiated of all the pairwise group comparisons among the 20 clusters.

### Principal coordinate analysis

**Fig 3:**
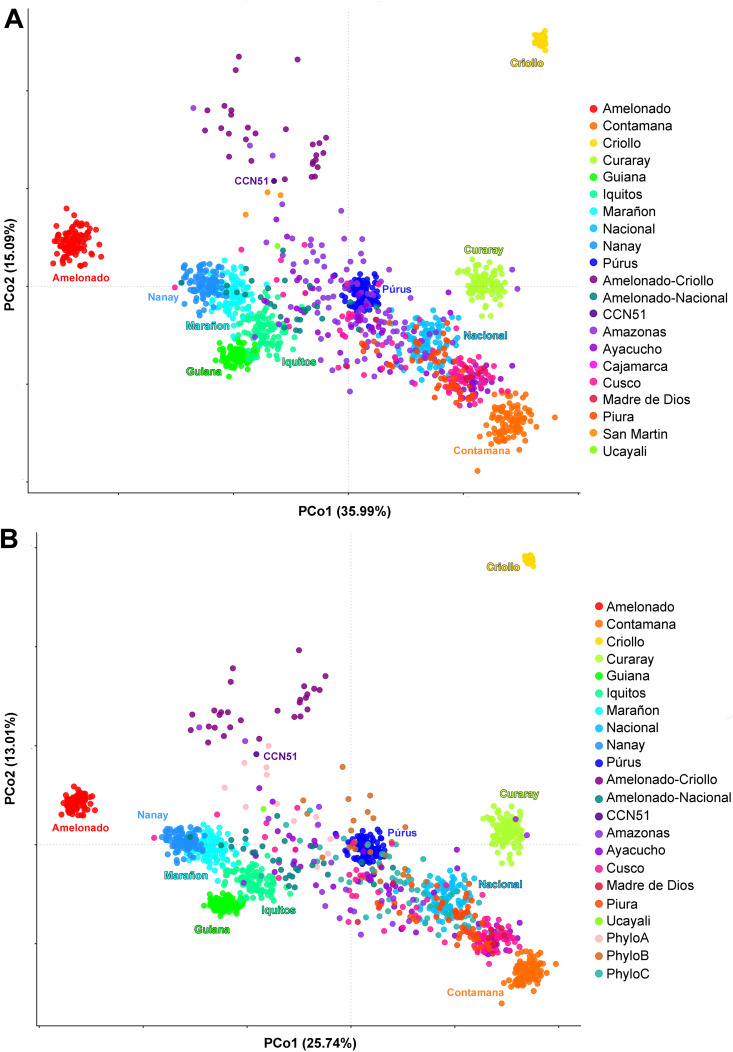
Principal coordinate analysis of indigenous cacao sampled in Peru based on (a) Departments in Peru and (b) using phylogenetic groups as the 10 reference populations of Motamayor et al. [8], those identified in Bustamante et al. [12] and recently collected departments.

The PCoA on the dataset of all individuals (Fig 3) showed a clear separation of the 10 genetic clusters of Motamayor et al. [8] but a varied and mixed distribution of the recently collected samples from Peru in this study. The Ayacucho, Cusco, Madre de Dios, and Piura samples were clustered between the Nacional and Contamana clusters. The Amazonas, Cusco, PhyloA, PhyloB and PhyloC had a more dispersed distribution along the middle diagonal of all four quadrants especially Amazonas and PhyloB samples. The single Ucayali sample was located in the quadrant diametrically opposed to the Contamana cluster. The CCN 51 sample had a very close overlap with a sample from the PhyloA sampling group.

**Fig 4:**
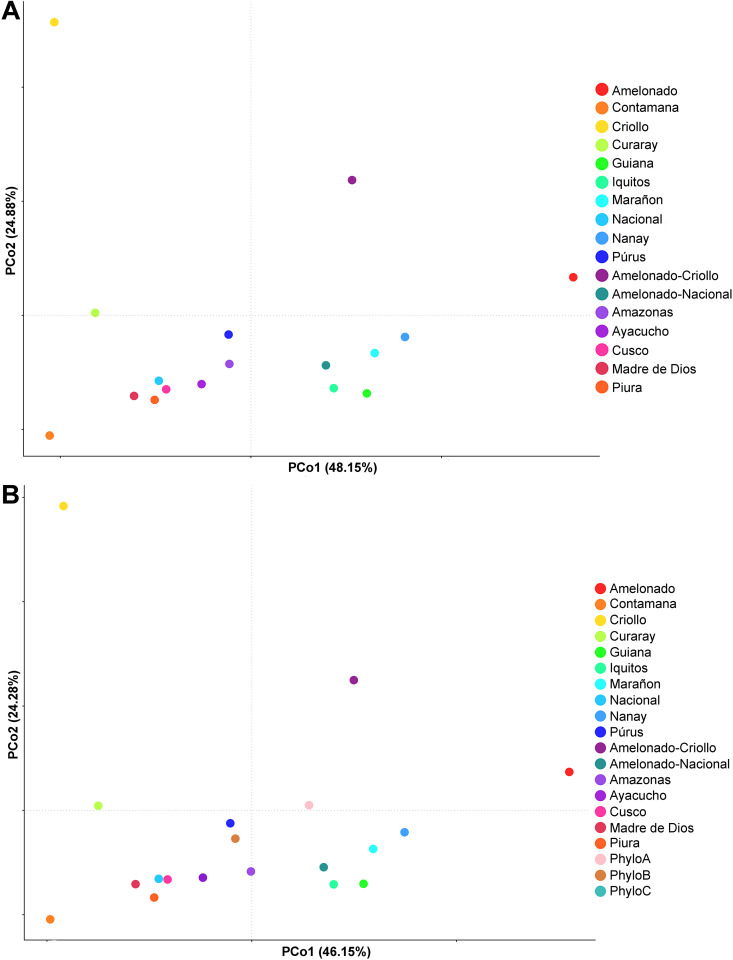
Principal coordinate analysis from Jost [19,20] D_est_ group differentiation statistic of indigenous cacao sampled in Peru based on (a) Departments in Peru and (b) using phylogenetic groups as the 10 reference populations of Motamayor et al. [8] and those identified in Bustamante et al. [12] and recently collected departments.

The PCoA on the Jost [21,219] D_est_ differentiation matrix (Fig 4) showed that the recently collected samples from Peru in this study were more related to the Upper Amazon groups especially Contamana, Curaray, Nacional, and Purús. The Cusco, Madre de Dios and Piura samples were closest to the Nacional group. The PhyloB group was closest to Purus. The PhyloC and Ayacucho groups overlapped each other and were between the

Nacional and Amazonas groups. The Amazonas group was found in the middle of the lower two quadrants midway between the Contamana/Nacional and the Marañon/Nanay clusters. The PhyloA group was most different from the other samples from Peru in this study being found in the same quadrant as the Amelonado group but towards the middle of the PCoA chart.

### Phylogeny

**Fig 5:**
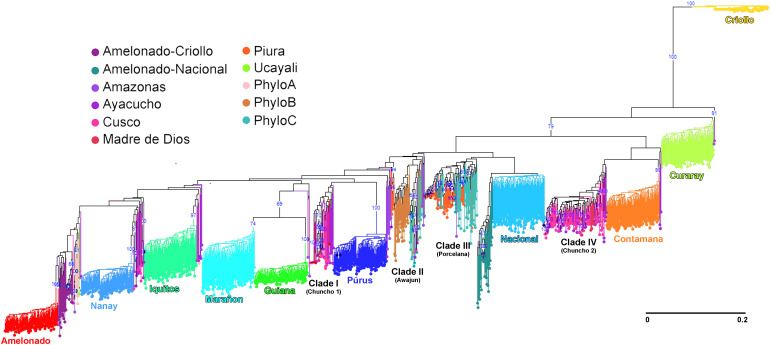
Cladogram of wild Peruvian cacao with 10 reference genetic clusters of cacaos. Clades I – IV represent putative new genetic clusters.

The cladogram (Fig 5) revealed that the newly collected samples from Peru were variably distributed among but not within the established 10 clades of Motamayor et al. [8]). The latter presented with 74%−100% bootstrapped linkages to the main tree except for Criollo (32%) and Nacional (0%). Four well-represented clades were formed by the samples collected under this study from Peru and contained 313 samples (80.3% of the 390 sampled trees; Table 3 and Fig 5). These four clades were found at Purús, mid-tree, Nacional and Contamana clusters and were identified as Clades I-IV containing 45, 52, 99 and 117 members respectively. Two samples (TCC22 and TCC23 from Amazonas) formed a sister clade with the Criollo and Curaray clades with 91% bootstrap support. The TCC22/TCC23 was the only clade with >50% bootstrap support. Although several subclades (most with two members) present throughout the nested clades of the samples from Peru had at least 50% bootstrap support, the main linkage for any of the umbrella clades of the nested samples had less than 50% bootstrap support.

**Table 3:** Sample composition of putative clusters of newly collected cacao trees from Peru based on phylogeny clustering. Samples collected from San Martin (3) and Ucayali (1) were absent from Clades I-IV.

Clade	Number of samples (% of samples for clade) collected from Department of						Total
	Amazonas	Ayacucho	Cajamarca	Cusco	Madre de Dios	Piura	
Clade I	0	16 (35.6%)	0	27 (60.0%)	2 (4.4%)	0	45
Clade II	47 (90.4%)	2 (3.8%)	0	2 (3.8%)	0	1 (1.9%)	52
Clade III	47 (47.5%)	0	1 (1.0%)	0	0	51 (51.5%)	99
Clade IV	0	38 (32.5%)	0	71 (60.7%)	8 (6.8%)	0	117
Total in Clade (% of total collected)	94 (72.3%)	56 (73.7%)	1 (100%)	100 (90.9%)	10 (100%)	52 (88.1%)	313 (80.3%)
Total Uniques Collected	130	76	1	110	10	59	386

Frequency distribution of the collected samples by Department and Clades I-IV is presented in Table 3. The collected samples in this study from Peru were mainly captured in these four clades except for the samples from San Martin (3) and Ucayali (1). These four samples were present between the Amelonado and Nanay clusters and as outgroups to the Amelonado-Criollo hybrids. The Amazonas samples were mainly present in Clade II (47 of 52; 90.4%) and Clade III (47 of 99; 47.5%). The Ayacucho samples were mainly present in Clade I (16 of 45; 35.6%) and Clade IV (38 of 117; 32.5%). The Cusco samples were mainly present in Clade I (27 of 45; 60.0%) and Clade IV (71 of 117; 60.7%). The Piura and Cajamarca samples were found in Clade III (51 of 99; 51.5%).

### Ancestry

The STRUCTURE output [27] showed optimal signature at K = 11 (S2 Fig) with alternative higher selections at K = 13 and K = 17 for the Phase I analysis of the 390 samples collected in this study in Peru with the simulated populations of Motamayor et al. [8]. However, the 10 reference populations of Motamayor et al. [8] were only properly resolved at K = 10 (15 of 20 runs) and at K = 11 (3 of 20 runs).

In contrast, each of the four separate Phase II ancestry clade analyses showed proper resolution of the 10 reference populations at K = 11 for 80%−100% of the 10 runs for a clade. Samples with high membership in the putative new genetic clusters are provided in Table 4. Using the Amelonado-Criollo individuals as a putative genetic group and running at K = 11 generated the same Amelonado-Criollo ancestry with the new population being present at very low levels (max = 0.0026) in a sample. However, using the Amelonado-Nacional individuals as a group and fitting 11 clusters gave a different result. The ancestry of these 28 individuals was separated into three groups – Amelonado-Newpop (1), Newpop only (7) and Nacional-Newpop (20).

**Table 4:** Membership allocation in new putative population based on clade clustering to guide ancestry analysis. ^1^Clades as identified from phylogenetic analysis in the current study; ^2^defined as correct allocation of the 10 reference genetic clusters of Motamayor et al. [8]; ^3^defined as having less than 5% membership in any of the 10 reference genetic clusters of Motamayor et al. [8].

Clade^1^	Number of members in clade	Number of successful runs of 10 iterations^2^	Number of members with 85% − 94% ancestry	Number of members with at least 95% ancestry	Number of pure group members^3^
Clade I	45	10	7	18	22
Clade II	52	10	7	20	24
Clade III	99	8	28	50	63
Clade IV	117	10	7	97	97

**Fig 6:**
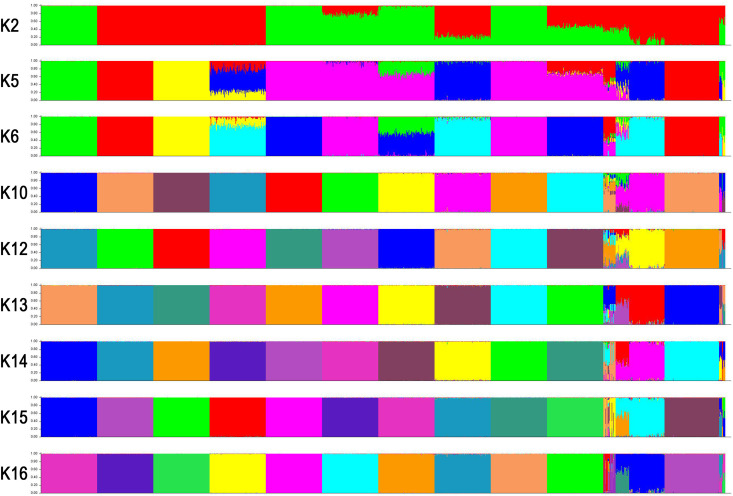
Representative ancestry plots of Phase III data analysis. Samples arrayed as simulated populations in Amelonado, Contamana, Criollo, Curaray, Guiana, Iquitos, Marañon, Nacional, Nanay, Púrus (100 individuals each); Clade I (n = 22); Clade II (n = 24); Clade III (n = 63); Clade IV (n = 97); Amelonado-Nacional (n = 5); CCN 51; Amelonado-Criollo (n = 5).

In Phase III ancestry analysis, the best fit number of clusters was K = 2 with a ΔK of 1415.99, nearly 1,400-fold higher than that at other clusters. The Evanno plot (S3 Fig) from K = 3–18 had a multimodal profile with highest peaks at K = 5, 12 and 14. At K = 2, the ten simulated populations were grouped as Amelonado/Guiana/Marañon/Nanay (Group 1) and Contamana/Criollo/Curaray (Group 2) (Fig 6). Iquitos, Nacional and Púrus were admixed from these two groups. Iquitos and Nacional had major contributions from Group 1 and Group 2 respectively while Púrus had similar contributions from each group. At K = 5, Clades III and IV were in the same genetic cluster as Nacional and Contamana respectively (Fig 6). Clades I and II were admixed with contributions from Clades IV and III respectively. Clades I and II remained predominantly admixed at K = 10, 12, 13 and 14 (S3 Table). The minimum number of clusters was K = 12 to capture the population structure in the dataset. The two new populations emerged from Clade IV (10 of 10 runs) and either from Clades I (4 of 10 runs), II (4 of 10 runs) and III (2 of 10 runs) respectively (S3 Table).

At K = 13, the new populations in Phase III analysis emerged either from Clades IV (14 of 15 runs), Clade I (11 of 15 runs), Clade III (10 of 15 runs), and Clade II (6 of 15 runs) in decreasing frequency of the Phase III analysis (S3 Table). In all cases, with Clade I as a new population, only nine of the 22 individuals were allocated to the new group, but the same nine individuals were consistently allocated. In the cases where Clade III was not a new population, it was allocated to the Nacional cluster containing pure Nacional members or pure Nacional members together with individuals admixed with Nacional and Clade II. However, CCN 51 was positioned as a new population with Clades III and IV as the other two new populations and with CCN 51 as the only pure Clade II member in four of the 15 runs. When CCN 51 was positioned as the only pure Clade II member, one and two individuals in the Amelonado-Criollo and Amelonado-Nacional references respectively consistently had an additional contribution from the new Clade II group. In the remaining 11 runs, the three new populations were derived from three combinations: Clades I, II & III; Clades I, II & IV; and Clades I, III & IV. The latter two were the most frequent (5 runs each).

At K = 14, the new populations in Phase III analysis emerged in all four clades (S3 Table). Clades III and IV had pure members in all 10 runs, Clade I had pure Clade I members and admixed members (Clade I/Clade III) in all 10 runs. Clade II was either all mixed as Clade II/Clade III (7 runs) or contained both mixed (Clade II/Clade III) and pure (18 of 24 members; 75%) Clade II members (3 of 10 runs). In the more frequent case of Clade II being all mixed, the ancestry of CCN 51 was ~ 99% Clade II and was the only individual with pure membership in Clade II. The Amelonado-Criollo individuals were as expected except for one (ICS 1) that exhibited 24.5% − 26.2% Clade II ancestry. The Amelonado-Nacional individuals also had Clade II ancestry. One member exhibited 11.8% − 12.3% Clade II ancestry and two others with low (1.2% − 3.3%) Clade II ancestry. In the less frequent case of Clade II having 75% pure Clade II members, the ancestry of CCN 51 had 71.6% − 86.7% Clade II ancestry with variable Amelonado (6% − 13%), Iquitos (3% − 8%), Nanay (3%), and Criollo (0% − 4.5%) ancestry. The Amelonado-Criollo references were as expected without Clade II ancestry but two or three Amelonado-Nacional references had Clade II ancestry. One Amelonado-Nacional member exhibited 11.5% − 17.1% Clade II ancestry and the other one or two individuals had 1.4% − 2.5% Clade II ancestry.

In Phase IV ancestry analysis, the CCN 51 sample and two other Peru samples (CCA015, TCC4) that were not in Clades I – IV, were predominantly positioned as the only pure members of Clade II. This occurred in 50% of the 10 iterations at K = 12, 92% of the 13 iterations at K = 15, and 100% of the 19 and 15 iterations at K = 13 and 14 respectively. The remaining runs gave an ancestral background of 45% Iquitos, 28% Criollo and 27% Amelonado (K = 12) or 85% CCN 51 group, 10% Clade II and 4% Iquitos (K = 15). Since only one sample matched to CCN 51 (CCA015) and the other sample (TCC 4) was different by 28–29 SNPs to the CCN 51 match group, the ancestry run was rechecked. The three members with CCN 51 ancestry were removed and a Phase V ancestry analysis conducted. Additional ancestry projects were undertaken as required to confirm ancestry estimates.

The choice of K was among 12, 13 or 14 clusters. At K = 12, there was a tendency to have many admixed samples in which a larger number of genetic clusters contributed to the admixture. Samples allocated to Clade I were all admixed from at least three groups at K = 13 but at K = 14 only 36.4% were admixed from at least three groups. Samples allocated to Clade III were all mixed with Nacional at K = 13 but became higher members in Clade III at K = 14 with the majority being assigned as pure members. Clade II presented a mix of Clades II/III at both K = 13 and K = 14 settings. Clade IV was consistently assigned as a new cluster at 12, 13 or 14 cluster settings. The option of having four new clusters was therefore chosen to fit the dataset to have the least number of admixed samples.

**Fig 7:**
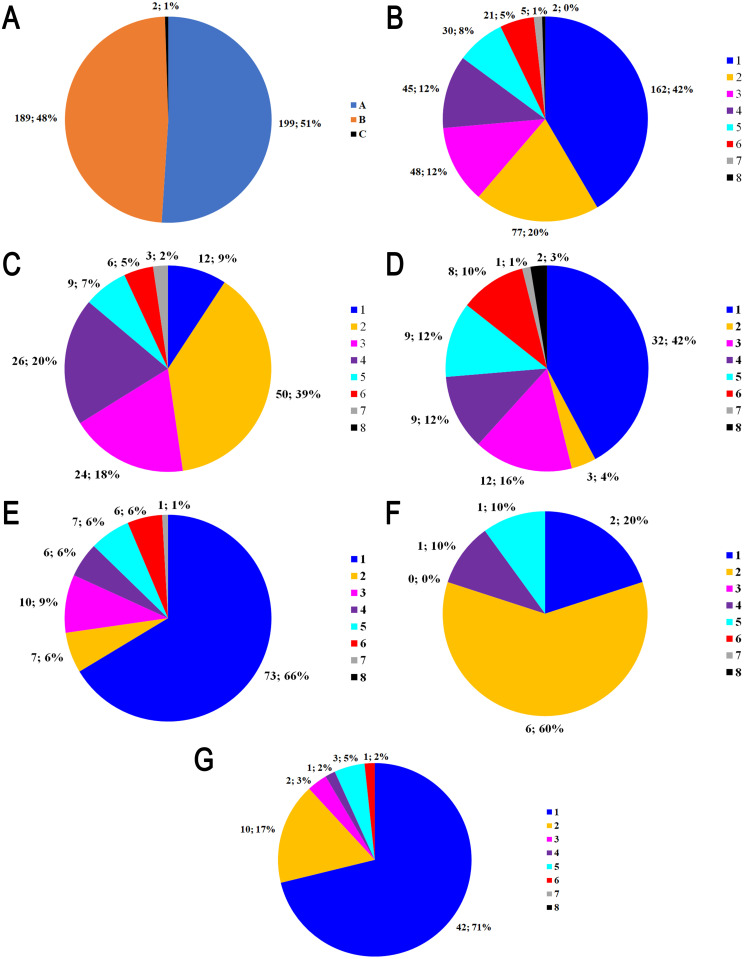
Number of genetic groups from ancestral combinations of cacao samples collected in Peru. A – Combinations in 390 samples based on **(A)** only Clades I-IV; **(B)** Clades I-IV with Motamayor et al. [8] reference genetic groups; and **(C)** Motamayor et al. [8] reference genetic groups in Peru; **(B-G)** – Combinations based on the number of genetic groups (max = 14; 10 from Motamayor et al. [8] and Clades I-IV); B, Peru (n = 390); C-G, Departments of Amazonas (n = 147), Ayacucho (n = 61), Cusco (n = 108), Madre de Dios (n = 10), and Piura (n = 59) respectively. The Cajamarca Department (n = 1) sample had only 1 genetic group; the San Martín Department (n = 3) had one sample with two genetic groups and two samples with four genetic groups; the Ucayali Department (n = 1) sample had five genetic groups.

At K = 14, a critical 3% minimum threshold was used to retain a group as contributing to the ancestral background of a sample. The 390 Peru samples had few samples with at least 10% Criollo (17 samples; 10% − 27.9%) or Nacional (17 samples; 10% − 27.6%) (Table 5). One sample (CPV 80) from La Convención, Cusco combined both of these ancestries. Members with at least 50% in any of the four new clusters were found in all Provinces except Huanta (Table 5). At 50% benchmark, Clade I and II members were predominantly found in La Convención (Cusco) and Utcubamba (Amazonas) respectively. Similarly, members with at least 50% Clade III ancestry were predominantly found in Utcubamba (Amazonas) and Huancabamba (Piura). Samples with 50% Clade IV ancestry were predominantly found in La Convención (Cusco) and La Mar (Ayacucho) (Table 5). At the critical minimal threshold of 3%, all but two samples had ancestral combinations from Clades I-IV with 199 samples (51%) having ancestral combinations among Clades I-IV only (Fig 7A). The majority of the samples in Peru were based on one (42%) or two (20%) genetic groups only (Fig 7B). Samples with only one genetic group predominated in the Departments of Ayacucho (42%, Fig 7D), Cusco (66%, Fig 7E) and Piura (71%, Fig 7G). Samples with only two genetic groups predominated in the Departments of Amazonas (39%, Fig 7C) and Madre de Dios (60%, Fig 7F). The TCC22/TCC23 samples, positioned as a sister clade, to the Curaray/Criollo clades had high Curaray ancestry (90.5% and 89.8% respectively) and very low Criollo ancestry (0.9% and 2.9% respectively).

**Table 5:** Ancestral allocation in the 390 samples collected in Peru.

		Min 10% in each group			Min 90% (50%) in each group			
Department	Province	Criollo	Nacional	Criollo & Nacional	I	II	III	IV
Amazonas (n = 130)	Bagua (n = 16)	1	0	0	0 (0)	0 (2)	2 (7)	0 (0)
	Condorcanqui (n = 32)	3	2	0	0 (1)	0 (4)	0 (8)	0 (0)
	Utcubamba (n = 82)	7	2	0	0 (0)	0 (20)	12 (47)	0 (0)
Ayacucho (n = 76)	Huanta (n = 3)	1	0	0	0 (0)	0 (0)	0 (0)	0 (0)
	La Mar (n = 73)	1	10	0	0 (8)	0 (1)	0 (0)	32 (39)
Cajamarca (n = 1)	Jaén (n = 1)	0	0	0	0 (0)	0 (0)	1 (1)	0 (0)
Cusco (n = 110)	La Convención (n = 110)	1	1	1	9 (15)	0 (0)	0 (0)	64 (75)
Madre de Dios (n = 10)	Inambari (n = 10)	0	0	0	0 (0)	0 (0)	0 (0)	4 (8)
Piura (n = 59)	Huancabamba (n = 59)	0	1	0	0 (0)	0 (0)	49 (57)	0 (0)
San Martín (n = 3)	Mariscal Cáceres (n = 3)	2	0	0	0 (0)	0 (2)	0 (0)	0 (0)
Ucayali (n = 1)	Atalaya (n = 1)	0	0	0	0 (0)	0 (1)	0 (0)	0 (0)

The ancestry of the CCN 51 variety was explored in additional STRUCTURE projects to complement the Clade and Phase I – IV ancestry analyses. There were three possible ancestral backgrounds – (a) 27% Amelonado, 28% Criollo and 43% Iquitos; (b) 99% Clade II and (c) 15% Amelonado, 13% Criollo, 25% Iquitos and 45% Clade II (Table 6). The Amelonado-Criollo-Iquitos option was found in projects involving all 10 reference populations either alone or in conjunction with individual clades (Clade I, clade III, Clade IV, Amelonado-Criollo, Amelonado-Nacional). CCN 51 presented as a pure Clade II member in projects involving (a) all 10 reference populations and the four clades at K = 14, (b) all 10 reference populations and only Clades II and III at K = 12, (c) three reference populations (Amelonado, Criollo, Iquitos) and Clades II and III at K = 5 and (d) four populations (Amelonado, Criollo, Iquitos, Nacional) and Clades II and III at K = 6. The Amelonado-Criollo-Iquitos-Clade II option was found when the 10 reference populations were used in conjunction only with Clade II.

**Table 6:** CCN 51 ancestry estimates under various modelling scenarios. ^1^Ancestry derived from STRUCTURE v2.3.4 [25], with burnin 200.00010,000, MCMC 300,000, admixture model and independent frequencies using (A) the run with the most positive lnP(D) or from (B) the LargeKGreedy algorithm of CLUMPP [28] implemented in StructureSelector [27] in independent ancestry simulation jobs using reference simulated populations. ^2^Datasets with 10 reference populations of Motamayor et al. [8] when K ≤ 10; with only one clade (Clades I-IV, Amel-Crio (28 Amelonado-Criollo), Amel-Naci (28 Amelonado-Nacional)); and variable reference samples. Phase III, IV has five each of the Amelonado-Criollo and Amelonado-Nacional references. Phase VI has 1 Amelonado-Criollo reference sample. Phase VIIa has three reference populations (Amelonado, Criollo, Iquitos), Clades II & III and one Amelonado-Criollo reference sample. Phase VIIb is Phase VIIa with the 10 reference populations. Clade II-III has four reference populations (Amelonado, Criollo, Iquitos, Nacional), Clades II & III and five each of the Amelonado-Criollo & Amelonado-Nacional reference samples. Clade IIa, b, c have the same dataset as Clade II; IIa is the same project as Clade II, IIb is an independent project at same settings, IIc is an independent project using 300,000 burnin and 600,000 MCMC settings.

Type^1^	Source^2^	K	Amelonado	Contamana	Criollo	Curaray	Guiana	Iquitos	Marañon	Nacional	Nanay	Púrus	Clade II
A	Prior	10	0.3116	0.0007	0.2739	0.0007	0.0011	0.4054	0.0018	0.0012	0.0027	0.0008	unused
A	Clade I	11	0.2745	0.0003	0.2828	0.0007	0.0011	0.4344	0.0015	0.0006	0.0027	0.0005	0.0007
A	Clade II	11	0.1546	0.0002	0.1293	0.0006	0.0008	0.2468	0.0021	0.0003	0.0092	0.0006	0.4555
A	Clade III	11	0.2732	0.0005	0.2829	0.0005	0.0011	0.4349	0.0019	0.0007	0.0034	0.0005	0.0005
A	Clade IV	11	0.2734	0.0003	0.283	0.0005	0.001	0.4345	0.0016	0.0006	0.0045	0.0005	0.0003
A	Amel-Crio	11	0.2749	0.0003	0.2816	0.0005	0.001	0.4334	0.0015	0.0009	0.0028	0.0005	0.0026
A	Amel-Naci	11	0.2653	0.0003	0.2818	0.0005	0.0011	0.4351	0.0017	0.0006	0.0030	0.0005	0.0101
A	Phase III	14	0.0013	0.0002	0.0007	0.0004	0.0007	0.0033	0.0010	0.0003	0.0025	0.0006	0.9878
A	Phase IV	14	0.0006	0.0003	0.0005	0.0004	0.0006	0.0015	0.0007	0.0003	0.0012	0.0005	0.9922
A	Phase VI	14	0.0001	0.0000	0.0001	0.0001	0.0001	0.0009	0.0001	0.0000	0.0002	0.0001	0.9820
A	Phase VIIa	5	0.0007	unused	0.0008	unused	unused	0.0161	unused	unused	unused	unused	0.9816
A	Phase VIIb	12	0.0007	0.0003	0.0007	0.0005	0.0001	0.0072	0.0010	0.000	0.0022	0.0013	0.9842
A	Clade II-III	6	0.0014	unused	0.0008	unused	unused	0.0045	unused	0.0004	unused	unused	0.9925
B	Amel-Crio	12	0.2746	0.0000	0.2817	0.0002	0.0012	0.4330	0.0018	0.0010	0.0030	0.0004	0.0030
B	Amel-Naci	12	0.2664	0.0000	0.2821	0.0002	0.0013	0.4350	0.0019	0.0009	0.0032	0.0006	0.0085
B	Clade IIa	11	0.1582	0.0000	0.1351	0.0001	0.0010	0.2504	0.0020	0.0000	0.0104	0.0010	0.4417
B	CladeIIb	11	0.1545	0.0000	0.1333	0.0000	0.0010	0.2483	0.0021	0.0001	0.0125	0.0010	0.4472
B	CladeIIc	11	0.1534	0.0000	0.1296	0.0001	0.0010	0.2433	0.0023	0.0002	0.0130	0.0010	0.4562

## Discussion

The genetic diversity and structure of cacao from its centre of diversity was examined in this study to confirm the existence of new genetic clusters other than those in the seminal work of Motamayor et al. [8]. This study supported the existence of other clusters as indicated from work on samples from Bolivia [10], Colombia [31], and Peru [11]. The possible limitations of these prior studies were mitigated by employing the use of both phylogenetic and ancestry analyses and the use of the former to guide reanalyses of the latter.

### Identity analysis

The most prominent feature of the dataset was the formation of 25 distinct match groups, overwhelmingly composed of duplicates found within the same department (22 groups). This pattern strongly suggests that dissemination of these cacao plants is highly focal, likely driven by local clonal expansion within communities or specific geographical areas [32]. The increasing frequency of duplicates from Piura (6), Ayacucho (9), and Cusco (13) indicates that these regions have a germplasm collection or farming system reliant on a limited number of propagated genotypes. An important finding is MG04, a single clone represented by eight (and potentially nine) individual samples, all from Cusco. A group of this size points to the existence of a highly favoured and widely disseminated cultivar in this region [33]. This prominent use of this genotype (Chuncho) resulted from the superior agronomic traits, such as high yield, disease resistance, and bean quality [34]. Additionally, the match, between sample CCA015 (Amazonas) and the reference sample CCN 51, confirmed the presence and cultivation of this commercially important clone in northern Peru (Amazonas). Conversely, the near-total lack of other matches implies that the vast majority of the sampled Peruvian cacao, particularly in Piura, Ayacucho, and Cusco, represents a genetically unique pool that is distinct from other countries and collections. The genetic uniqueness of most Peruvian material, barring the introduction of CCN 51, underscores the value of this germplasm as a unique genetic resource for breeding programs [35].

### Clustering

The results revealed a complex landscape characterized by deep ancestral divisions, widespread admixture among newly collected populations, and the identification of unique genetic resources. There was significant differentiation (P = 0.001–0.015) among all 20 genetic clusters indicative of the genetic and geographic separation of these groups. The primary germplasm groups of Motamayor et al. [8] exhibited relatively low D_est_ differentiation among the Amelonado/Guiana/Iquitos/Marañon/Nanay clusters which was supported by ancestral grouping at K = 2 indicative that this may be a metapopulation in cacao. The other K = 2 ancestral grouping was Contamana/Criollo/Curaray/Nacional but Criollo was well separated from these groups, suggesting that Criollo could have been isolated or undergone a bottleneck from a Contamana/Curaray/Nacional metapopulation. These two metapopulations with an intermediary Púrus group are also supported by the current and earlier [8,36,37] phylogenetic results with Fouet et al. [37] suggesting that Criollo was derived from Curaray. Guiterrez et al [38] also reported two genetic clusters in cacao. The 10 primary germplasm groups were established as distinct genetic clusters [8,37,39] with support from private microsatellite alleles [8,37]. However, the current study and Bustamante et al. [12] did not find any private alleles, which may be due to the limited number of markers and different maker systems used in these studies.

The new putative phylogenetic clusters demonstrated a variable differentiation from each other and the established clusters of Motamayor et al. [8] supporting their use as distinct clusters. The Madre de Dios group was most differentiated from the reference populations and the phylogenetic groups in the Peru samples of this study, suggesting a unique genetic composition, potentially with a higher proportion of one or more ancestral genotypes [38]. Conversely, Ayacucho, Cusco, and Madre de Dios showed remarkably little genetic divergence from each other (D_est_ = 0.010–0.030). This near-panmixia indicates extensive gene flow and/or shared ancestry among these Departments, likely facilitated by human-mediated movement of planting material [40]. Additionally, the Amazonas group appears to be a genetically central or admixed population. Its closeness to diverse clusters including Iquitos, Nacional, and the Phylo groups suggests it may contain a high level of the ancestral genetic diversity from which other types were derived [14]. Overall, the current study demonstrates that finer-scale population structure exists in Peru [12]. This refines our understanding of the cacao genetic landscape in Peru, moving beyond the broad categories to identify region-specific genetic signatures. While firmly rooted in the Upper Amazonian gene pool, it has been shaped by processes of admixture and regional differentiation, resulting in a spectrum of diversity from the highly admixed (Amazonas, Ayacucho, Cusco) to highly distinct (Madre de Dios, PhyloA).

The robust and consistent emergence of four new genetic clusters (Clades I-IV) derived from the Peruvian samples are not random assemblages but likely arise from specific, well-defined ancestral backgrounds within the Upper Amazonian complex. Briefly, clade IV demonstrated the strongest signal, consistently emerging as a distinct cluster across nearly all runs and K-values. This suggested that it is representative of a cohesive, genetically distinct population within Peru, likely derived from a Contamana-related ancestry. Clades I, II, and III showed a more variable pattern, sometimes emerging as distinct and other times being resolved as admixed with each other or with established clusters like Nacional (Clade III). This indicates that these groups are more recently derived or have experienced more ongoing gene flow, but still possess a unique genetic signature at higher K-values [14]. The geographic patterning of these clusters is also clear and significant. The concentration of Clade III in Huancabamba (Piura) and Utcubamba (Amazonas), Clade IV in La Convención (Cusco) and La Mar (Ayacucho), and Clades I and II in specific provinces, provides strong evidence for local adaptation or founder effects followed by regional propagation of these distinct genetic types [33]. Accordingly, based on their distinct geographic patterning and local predominance, these clades are named as follows. Clades I and IV are named Clade Chuncho 1 and Clade Chuncho 2, respectively, after the local name for cacao from Cusco. Clade II is named Clade Awajun after the predominant presence of this indigenous community in Amazonas. Clade III is named Clade Porcelana after the local name for cacao from Piura.

In the current study, samples were collected from eight Departments in Peru. The cacao trees from these Departments were variably mixed containing both pure members of new populations as well as admixed samples with variable composition of the ten reference populations of Motamayor et al. [8] and the new Peruvian populations. The Huancabamba (Piura) and Utcubamba (Amazonas) samples were preferentially allocated to Clade Porcelana. The samples from Madre de Dios were grouped in Clade Chuncho 2 together with samples primarily located in Cusco. The separation of a Piura group from a Cusco group is therefore supported. However, distinct clusters for Madre de Dios and Cusco samples could not be supported which differed from [11].

The findings of this study suggest that while the cacao germplasm is genetically related across different departments, each region harbors its own distinct genetic composition. Since the Nacional group and Clades Awajun and Porcelana appear to be related based on ancestry allocation and phylogeny, samples with high membership in either of the latter two groups are good candidates for fine flavour and high quality. It is recommended that the samples with highest membership in any of the four new groups be assessed for their quality and sensory attributes. These samples provide a base which will improve the position of Peru in the fine cocoa industry.

### CCN 51 ancestry

The current study also revealed that the increasingly important cultivar CCN 51 [41,42] had cryptic ancestry based on the composition of the dataset used for ancestry analysis in STRUCTURE. Puechmaille [43] cautioned that uneven sampling led to unreliable ancestry estimates in STRUCTURE. Our study suggested as well that the composition in terms of the ancestral groups present is also important. The presence of members with very high membership in Clade Awajun when only this Clade was the additional group and the resultant absence of pure members when Clade Porcelana was also present is further supporting evidence that the types of genetic clusters present is important. Forcing a new additional group without any such group present gave the Iquitos/Amelonado/Criollo ancestry for CCN 51 when either the Amelonado-Criollo or Amelonado-Nacional was used as the additional population at K = 11. Furthermore, there was no clear signal for the 11^th^ population when the Amelonado-Criollo clade was used, but when the Amelonado-Nacional clade was used, the new population presented within the Nacional population. The current study therefore supports the use of independent STRUCTURE jobs and the appropriate use of known populations to correctly infer the ancestral admixture in cacao plants. The results of this study also suggests that the accessions with Nacional ancestry in the ICGT should be reassessed with the four new populations identified in this study to get a more reliable estimate of their ancestral background.

According to Boza et al. [41] CCN 51was derived from a triple cross as (IMC 67 × ICS 95) ×“Canelo” (Oriente 1). The latter parent is unknown and the ancestry of CCN 51 was reported by Boza et al. [41] and reviewed by Jaimez et al. [42.]. Boza et al. [41] estimated the admixture in CCN 51 to be 45.4% Iquitos, 22.2% Criollo, 21.5% Amelonado, 3.9% Contamana, 2.5% Purús, 2.1% Marañon and 1.1% Nacional. However, this ancestral background of CCN 51 was queried, as it is a suspected suboptimal run [8] and only based on the 10 populations of Motamayor et al. [8]. The accession IMC 67 has a 100% Iquitos background and ICS 95 is 47% Amelonado and 52% Criollo. This suggests that CCN 51 should have at least 25% Iquitos ancestry, 12% Amelonado ancestry and 13% Criollo ancestry along with the unknown background of Oriente 1 from Pastaza, Ecuador. The combination dataset of the 10 reference populations of Motamayor et al. [8] with Clade Awajun was the only dataset to confirm this expected background with estimates of 15% Amelonado, 13% Criollo, 25% Iquitos and 45% Clade Awajun. The “Canelo” parent is therefore likely to have very high membership (at least 90%) in the Clade Awajun population. The cryptic ancestry of CCN 51 revealed in this study suggests that undetected ancestral contributions may be present in some cacao germplasm and that this may be a result of sample ascertainment bias in STRUCTURE. The ancestry estimates are dependent on the provided mix of genetic lineages and the unknown samples for which ancestry is to be determined. This finding is in alignment with Puechmaille [43], Meirmans [44] and Toyama [45] but has not been previously reported in cacao. We suggest that multiple approaches be used when inferring ancestry from empirical studies and that ancestry estimates be viewed with caution in cacao.

## Supporting information

S1 Table Sampling location details of 390 cacao trees in Peru. (DOCX)

S2 Table SNPs of 390 cacao trees from Peru. (XLSX)

S3 Table Summary details of iterations at different cluster assignment with Phase III dataset. Phase III dataset has the 10 simulated populations of Motamayor et al. (2008), members from four phylogenetic clades, five Amelonado-Nacional accessions, CCN 51 and five Amelonado-Criollo accessions.
(DOCX)

S1 Fig Workflow summarising graphically the methodology. (DOCX)

S2 Fig Preliminary Evanno plot of samples collected in Peru (390) with 10 simulated populations. (DOCX)

S3 Fig Evanno plot based on dataset of simulated population clusters of Motamayor et al. [8] and pure group members in four phylogenetic clades. (DOCX)
