# Supplementary material for: Genetic structure of traditional cacao reveals four new genetic lineages in indigenous Amazonian sites in Peru
Source: PLoS One. 2026 Jul 6;21(7):e0351690. doi: 10.1371/journal.pone.0351690 (PMC13336180; doi:10.1371/journal.pone.0351690)
Supplement: S1 Table — (DOCX) [file pone.0351690.s001.docx]

**Genetic structure of traditional cacao reveals four new genetic lineages in indigenous Amazonian sites in Peru**

**Supplemental Table S1.** Sampling location details of 390 cacao trees in Peru.

| **Sample** | **Department** | **Province** | **Sector** | **UTM-X** | **UTM-Y** | **Collection date** |
| --- | --- | --- | --- | --- | --- | --- |
| INDES021 | Amazonas | Bagua | Santa Ana | 787203 | 9370435 | 2018 |
| INDES023 | Amazonas | Bagua | Santa Ana | 787192 | 9370422 | 2018 |
| INDES033 | Amazonas | Bagua | Lluhuana | 788281 | 9372123 | 2018 |
| INDES034 | Amazonas | Bagua | Lluhuana | 787570 | 9371144 | 2018 |
| INDES038 | Amazonas | Bagua | Copallin | 786789 | 9372113 | 2018 |
| INDES060 | Amazonas | Bagua | Copallin | 786846 | 9377122 | 2018 |
| INDES068 | Amazonas | Bagua | Pan de Azucar | 789227 | 9372471 | 2018 |
| INDES069 | Amazonas | Bagua | Pan de Azucar | 789342 | 9372455 | 2018 |
| INDES070 | Amazonas | Bagua | Lluhuana | 787756 | 9371938 | 2018 |
| INDES077 | Amazonas | Bagua | Tañuspe | 779257 | 9378941 | 2018 |
| INDES078II | Amazonas | Bagua | Tañuspe | 779260 | 9378861 | 2018 |
| INDES079I | Amazonas | Bagua | Tañuspe | 779183 | 9378799 | 2018 |
| INDES095 | Amazonas | Bagua | Peca Palacios | 778679 | 9372344 | 2018 |
| INDES096 | Amazonas | Bagua | Peca Palacios | 778669 | 9372343 | 2018 |
| INDES100 | Amazonas | Bagua | Pan de Azucar | 798067 | 9362827 | 2018 |
| INDES147 | Amazonas | Bagua | Pan de Azucar | 798067 | 9362827 | 2018 |
| CCA004 | Amazonas | Condorcanqui | C.N. Pumpu | 795263 | 9453932.5 | 2020 |
| CCA009 | Amazonas | Condorcanqui | Mochinguis | 190100 | 9606033 | 2020 |
| CCA013 | Amazonas | Condorcanqui | Juan Velasco | 183492 | 9489705 | 2020 |
| CCA015 | Amazonas | Condorcanqui | Chapiza | 197453 | 9587437 | 2020 |
| CCA018 | Amazonas | Condorcanqui | Candungos | 184075 | 9618414 | 2020 |
| CCA019 | Amazonas | Condorcanqui | Candungos 1 | 183477 | 9616432 | 2020 |
| CCA021 | Amazonas | Condorcanqui | Soledad | 188486 | 9611208 | 2020 |
| CCA022 | Amazonas | Condorcanqui | Soledad | 188486 | 9611208 | 2020 |
| CCA026 | Amazonas | Condorcanqui | C.N. Tsamaren | 790556.7 | 9426589.4 | 2020 |
| TCC_01 | Amazonas | Condorcanqui | Pampa Hermosa | 183698.2 | 9492109.516 | 2021 |
| TCC_02 | Amazonas | Condorcanqui | Pampa Hermosa | 183695.2 | 9492084.935 | 2021 |
| TCC_03 | Amazonas | Condorcanqui | Puerto Galilea | 193746.4 | 9555823.583 | 2021 |
| TCC_04 | Amazonas | Condorcanqui | Villa Gonzalo | 195097.301 | 9564542.528 | 2021 |
| TCC_05 | Amazonas | Condorcanqui | Villa Gonzalo | 195134.2 | 9564582.599 | 2021 |
| TCC_06 | Amazonas | Condorcanqui | Huabal | 195250.3 | 9559374.623 | 2021 |
| TCC_07 | Amazonas | Condorcanqui | Nieva | 181738.801 | 9491447.033 | 2021 |
| TCC_08 | Amazonas | Condorcanqui | Nieva | 181717.2 | 9491437.761 | 2021 |
| TCC_09 | Amazonas | Condorcanqui | Huampami | 815860.3 | 9508555.159 | 2021 |
| TCC_10 | Amazonas | Condorcanqui | Kuzumatak | 820645.699 | 9456744.087 | 2022 |
| TCC_11 | Amazonas | Condorcanqui | Kuzumatak | 820679.5 | 9456734.754 | 2022 |
| TCC_12 | Amazonas | Condorcanqui | Kuzumatak | 820750.199 | 9456669.811 | 2022 |
| TCC_13 | Amazonas | Condorcanqui | Kuzumatak | 820801.9 | 9456497.591 | 2022 |
| TCC_14 | Amazonas | Condorcanqui | Tokio | 820254.142 | 9459622.229 | 2022 |
| TCC_15 | Amazonas | Condorcanqui | Tsuan | 820597.136 | 9456931.788 | 2022 |
| TCC_16 | Amazonas | Condorcanqui | Najaim Paraiso | 825569.2 | 9457192.836 | 2022 |
| TCC_17 | Amazonas | Condorcanqui | Pampa Hermosa | 183704.4 | 9492097.146 | 2022 |
| TCC_18 | Amazonas | Condorcanqui | Pampa Hermosa | 183689 | 9492094.096 | 2022 |
| TCC_19 | Amazonas | Condorcanqui | Pampa Hermosa | 183692.1 | 9492087.911 | 2022 |
| TCC_20 | Amazonas | Condorcanqui | Pampa Hermosa | 183729.127 | 9492088.058 | 2022 |
| TCC_21 | Amazonas | Condorcanqui | Ebron | 184162.401 | 9488647.912 | 2022 |
| TCC_22 | Amazonas | Condorcanqui | Parque Nacional Ichigkat Muja - Cordillera del Condor | 184325 | 9665707 | 2022 |
| TCC_23 | Amazonas | Condorcanqui | Parque Nacional Ichigkat Muja - Cordillera del Condor | 184355 | 9665935 | 2022 |
| CAP010 | Amazonas | Utcubamba | La Concordia | 788648 | 9368390 | 2018 |
| CAP028 | Amazonas | Utcubamba | La Concordia | 788629 | 9368870 | 2018 |
| CAP032 | Amazonas | Utcubamba | Cajaruro | 788563 | 9368822 | 2018 |
| CAP037_A | Amazonas | Utcubamba | Cajaruro | 793950 | 9368950 | 2018 |
| CAP040 | Amazonas | Utcubamba | Cajaruro | 793934 | 9366210 | 2018 |
| CAP041 | Amazonas | Utcubamba | Diamante Bajo | 793930 | 9366195 | 2018 |
| CAP043 | Amazonas | Utcubamba | San Jose Bajo | 793862 | 9366213 | 2018 |
| CAP044 | Amazonas | Utcubamba | Diamante Bajo | 793894 | 9366218 | 2018 |
| CAP046 | Amazonas | Utcubamba | Cajaruro | 793925 | 9366173 | 2018 |
| CAP047 | Amazonas | Utcubamba | Cajaruro | 793900 | 9366213 | 2018 |
| CAP050 | Amazonas | Utcubamba | Cajaruro | 793591 | 9367112 | 2018 |
| CAP062 | Amazonas | Utcubamba | Quebrada Seca | 779009 | 9367468 | 2018 |
| CAP074A | Amazonas | Utcubamba | Cajaruro | 787302 | 9369685 | 2018 |
| CAP083 | Amazonas | Utcubamba | Quebrada Seca | 779558 | 9367830 | 2018 |
| CAP086 | Amazonas | Utcubamba | San Jose Bajo | 787175 | 9368448 | 2018 |
| CAP092 | Amazonas | Utcubamba | Cajaruro | 793670 | 9366982 | 2018 |
| CAP102 | Amazonas | Utcubamba | San Jose Bajo | 787236 | 9368519 | 2018 |
| CAP103_A | Amazonas | Utcubamba | San Jose Bajo | 787230 | 9368540 | 2018 |
| CAP104 | Amazonas | Utcubamba | San Jose Bajo | 787236 | 9368478 | 2018 |
| CAP105_A | Amazonas | Utcubamba | La Concordia | 787987 | 9368059 | 2018 |
| CAP107A | Amazonas | Utcubamba | Cajaruro | 794708 | 9369438 | 2018 |
| CAP130 | Amazonas | Utcubamba | Manantial | 788854 | 9369551 | 2018 |
| CAP131 | Amazonas | Utcubamba | San Jose Bajo | 787194 | 9368461 | 2018 |
| INDES001 | Amazonas | Utcubamba | La Concordia | 831352 | 9243625 | 2018 |
| INDES002 | Amazonas | Utcubamba | La Concordia | 788958 | 9368566 | 2018 |
| INDES003 | Amazonas | Utcubamba | La Concordia | 788997 | 9368586 | 2018 |
| INDES004 | Amazonas | Utcubamba | La Concordia | 788671 | 9368556 | 2018 |
| INDES005 | Amazonas | Utcubamba | La Concordia | 788870 | 9369584 | 2018 |
| INDES006 | Amazonas | Utcubamba | El Chalan | 787894 | 9369168 | 2018 |
| INDES007 | Amazonas | Utcubamba | El Chalan | 787898 | 9369180 | 2018 |
| INDES009 | Amazonas | Utcubamba | El Chalan | 787898 | 9369126 | 2018 |
| INDES010 | Amazonas | Utcubamba | El Chalan | 787776 | 9369072 | 2018 |
| INDES011 | Amazonas | Utcubamba | El Chalan | 787792 | 9369112 | 2018 |
| INDES012 | Amazonas | Utcubamba | El Chalan | 793633 | 9366965 | 2018 |
| INDES013 | Amazonas | Utcubamba | El Limoncito | 793718 | 9366968 | 2018 |
| INDES014 | Amazonas | Utcubamba | El Limoncito | 793728 | 9366961 | 2018 |
| INDES015 | Amazonas | Utcubamba | El Limoncito | 793739 | 9366957 | 2018 |
| INDES016 | Amazonas | Utcubamba | El Limoncito | 793741 | 9366973 | 2018 |
| INDES017 | Amazonas | Utcubamba | La Cruz | 786963 | 9370270 | 2018 |
| INDES019 | Amazonas | Utcubamba | La Cruz | 786938 | 9370273 | 2018 |
| INDES024 | Amazonas | Utcubamba | La Concordia | 788555 | 9368399 | 2018 |
| INDES025 | Amazonas | Utcubamba | La Cruz | 786935 | 9370012 | 2018 |
| INDES026 | Amazonas | Utcubamba | Santa Ana | 786835 | 9369973 | 2018 |
| INDES027 | Amazonas | Utcubamba | La Cruz | 786945 | 9369993 | 2018 |
| INDES028 | Amazonas | Utcubamba | El Limoncito | 793600 | 9367107 | 2018 |
| INDES029 | Amazonas | Utcubamba | Jahuanga | 771472 | 9361437 | 2018 |
| INDES030 | Amazonas | Utcubamba | Jahuanga | 711468 | 9361430 | 2018 |
| INDES031 | Amazonas | Utcubamba | Jahuanga | 770538 | 9361358 | 2018 |
| INDES032 | Amazonas | Utcubamba | Quebrada Seca | 779564 | 9367833 | 2018 |
| INDES035 | Amazonas | Utcubamba | Quebrada Seca | 779584 | 9367821 | 2018 |
| INDES047 | Amazonas | Utcubamba | Guinguillo | 773015 | 9359963 | 2018 |
| INDES048 | Amazonas | Utcubamba | Guinguillo | 773018 | 9359976 | 2018 |
| INDES049 | Amazonas | Utcubamba | Guinguillo | 772833 | 9359809 | 2018 |
| INDES050 | Amazonas | Utcubamba | Diamante Bajo | 794447 | 9366031 | 2018 |
| INDES051 | Amazonas | Utcubamba | Diamante Bajo | 794446 | 9366639 | 2018 |
| INDES052 | Amazonas | Utcubamba | Diamante Bajo | 794441 | 9366649 | 2018 |
| INDES053 | Amazonas | Utcubamba | Diamante Bajo | 794453 | 9366666 | 2018 |
| INDES055 | Amazonas | Utcubamba | Diamante Bajo | 794476 | 9366699 | 2018 |
| INDES061 | Amazonas | Utcubamba | Santa Ana | 786427 | 9371077 | 2018 |
| INDES063 | Amazonas | Utcubamba | Naranjos Alto | 793806 | 9365734 | 2018 |
| INDES064 | Amazonas | Utcubamba | Naranjos Alto | 792251 | 9364133 | 2018 |
| INDES065 | Amazonas | Utcubamba | Naranjos Alto | 792308 | 9364147 | 2018 |
| INDES066 | Amazonas | Utcubamba | Naranjos Alto | 792346 | 9364181 | 2018 |
| INDES072 | Amazonas | Utcubamba | La Cruz | 786910 | 9370292 | 2018 |
| INDES073 | Amazonas | Utcubamba | Llunchicate | 801517 | 9364791 | 2018 |
| INDES074 | Amazonas | Utcubamba | El Limoncito | 801491 | 9364914 | 2018 |
| INDES075 | Amazonas | Utcubamba | Llunchicate | 801505 | 9364904 | 2018 |
| INDES076 | Amazonas | Utcubamba | José Olaya | 798295 | 9363632 | 2018 |
| INDES080 | Amazonas | Utcubamba | Diamante Bajo | 794303 | 9367139 | 2018 |
| INDES081 | Amazonas | Utcubamba | Diamante Bajo | 794270 | 9367134 | 2018 |
| INDES083 | Amazonas | Utcubamba | Diamante Bajo | 794282 | 9367102 | 2018 |
| INDES084 | Amazonas | Utcubamba | Diamante Bajo | 794315 | 9367127 | 2018 |
| INDES086 | Amazonas | Utcubamba | Diamante Bajo | 795932 | 9368212 | 2018 |
| INDES087 | Amazonas | Utcubamba | Diamante Bajo | 795844 | 9368227 | 2018 |
| INDES088 | Amazonas | Utcubamba | Diamante Bajo | 795886 | 9368101 | 2018 |
| INDES089 | Amazonas | Utcubamba | Diamante Bajo | 795878 | 9368188 | 2018 |
| INDES090 | Amazonas | Utcubamba | Diamante Bajo | 795951 | 9368212 | 2018 |
| INDES091 | Amazonas | Utcubamba | José Olaya | 797640 | 9365621 | 2018 |
| INDES092 | Amazonas | Utcubamba | Quebrada Seca | 779117 | 9367514 | 2018 |
| INDES093 | Amazonas | Utcubamba | Quebrada Seca | 779114 | 9367500 | 2018 |
| INDES094 | Amazonas | Utcubamba | Quebrada Seca | 779112 | 9367488 | 2018 |
| INDES099 | Amazonas | Utcubamba | EL TIGRE | 798067 | 9362827 | 2018 |
| CPV64 | Ayacucho | Huanta | Llochegua | 619170.353 | 8623771.382 | 2022 |
| VRAE81 | Ayacucho | Huanta | Sivia | 622692 | 8617181 | 1999 |
| VRAE86 | Ayacucho | Huanta | Sivia | 620971 | 8625423 | 1999 |
| CER01 | Ayacucho | La Mar | Chungui | 666847 | 8549054 | 2022 |
| CER03 | Ayacucho | La Mar | Anchihuay | 650315 | 8577235 | 2022 |
| CHMM01_P1 | Ayacucho | La Mar | Anco | 662779 | 8557734 | 2018 |
| CHMM02 | Ayacucho | La Mar | Chungui | 666807 | 8549249 | 2018 |
| CHMM03 | Ayacucho | La Mar | Chungui | 666927 | 8547326 | 2018 |
| CHMM04 | Ayacucho | La Mar | Chungui | 666941 | 8549638 | 2018 |
| CHMM05 | Ayacucho | La Mar | Chungui | 664853 | 8549765 | 2018 |
| CHMM06 | Ayacucho | La Mar | Chungui | 666920 | 8549630 | 2018 |
| CHMM08 | Ayacucho | La Mar | Chungui | 666804 | 8549247 | 2018 |
| CHMM09 | Ayacucho | La Mar | Chungui | 666800 | 8549250 | 2018 |
| CHMM10 | Ayacucho | La Mar | Chungui | 666810 | 8549235 | 2018 |
| CHMM13 | Ayacucho | La Mar | Chungui | 667574 | 8550035 | 2018 |
| CHMM14 | Ayacucho | La Mar | Chungui | 666801 | 8549315 | 2018 |
| CHMM15 | Ayacucho | La Mar | Anco | 661456 | 8557980 | 2018 |
| CHMM16 | Ayacucho | La Mar | Chungui | 667213 | 8549763 | 2018 |
| CMM01 | Ayacucho | La Mar | Ayna | 633620 | 8601103 | 2018 |
| CPV02_P1 | Ayacucho | La Mar | Anco | 662091.83 | 8551039.87 | 2021 |
| CPV02_P2 | Ayacucho | La Mar | Anco | 662098.98 | 8551074.9 | 2021 |
| CPV02_P3 | Ayacucho | La Mar | Anco | 662118.87 | 8551064.6 | 2021 |
| CPV02_P4 | Ayacucho | La Mar | Anco | 662139.54 | 8551058.62 | 2021 |
| CPV02_P5 | Ayacucho | La Mar | Anco | 662028.61 | 8551075.98 | 2021 |
| CPV03 | Ayacucho | La Mar | Anco | 662324.621 | 8557402.19 | 2022 |
| CPV04 | Ayacucho | La Mar | Anco | 662330.229 | 8557396.736 | 2022 |
| CPV05 | Ayacucho | La Mar | Anco | 662858.156 | 8557039.838 | 2022 |
| CPV06 | Ayacucho | La Mar | Anco | 662951.61 | 8557052.007 | 2022 |
| CPV07 | Ayacucho | La Mar | Anco | 662953.812 | 8557057.525 | 2022 |
| CPV08 | Ayacucho | La Mar | Anco | 662958.474 | 8557057.165 | 2022 |
| CPV09 | Ayacucho | La Mar | Anco | 662936.122 | 8555902.263 | 2022 |
| CPV10 | Ayacucho | La Mar | Anco | 662940.67 | 8555901.24 | 2022 |
| CPV11 | Ayacucho | La Mar | Anco | 662855.282 | 8555913.583 | 2022 |
| CPV12 | Ayacucho | La Mar | Chungui | 671057.999 | 8542430.005 | 2022 |
| CPV13 | Ayacucho | La Mar | Chungui | 671059.071 | 8542431.547 | 2022 |
| CPV14 | Ayacucho | La Mar | Chungui | 671060.142 | 8542432.801 | 2022 |
| CPV15 | Ayacucho | La Mar | Chungui | 671060.26 | 8542434.349 | 2022 |
| CPV16 | Ayacucho | La Mar | Chungui | 671061.332 | 8542435.891 | 2022 |
| CPV17 | Ayacucho | La Mar | Chungui | 671061.507 | 8542432.693 | 2022 |
| CPV18 | Ayacucho | La Mar | Chungui | 671061.069 | 8542431.966 | 2022 |
| CPV19 | Ayacucho | La Mar | Chungui | 667424.012 | 8550081.517 | 2022 |
| CPV20 | Ayacucho | La Mar | Chungui | 667440.556 | 8550091.705 | 2022 |
| CPV21 | Ayacucho | La Mar | Chungui | 667443.056 | 8550092.685 | 2022 |
| CPV22 | Ayacucho | La Mar | Chungui | 667444.1 | 8550103.741 | 2022 |
| CPV23 | Ayacucho | La Mar | Chungui | 667436.707 | 8550047.366 | 2022 |
| CPV24 (CPV24_P1) | Ayacucho | La Mar | Chungui | 667535.742 | 8550017.887 | 2022 |
| CPV25 | Ayacucho | La Mar | Chungui | 667598.11 | 8549592.484 | 2022 |
| CPV26 | Ayacucho | La Mar | Chungui | 664788.685 | 8550262.12 | 2022 |
| CPV27 | Ayacucho | La Mar | Chungui | 664792.35 | 8550258.669 | 2022 |
| CPV28 | Ayacucho | La Mar | Chungui | 664797.212 | 8550255.653 | 2022 |
| CPV29 | Ayacucho | La Mar | Chungui | 664805.259 | 8550259.476 | 2022 |
| CPV30 | Ayacucho | La Mar | Chungui | 664812.09 | 8550277.467 | 2022 |
| CPV31 | Ayacucho | La Mar | Chungui | 664836.202 | 8549762.024 | 2022 |
| CPV32 | Ayacucho | La Mar | Anco | 663538.106 | 8550219.837 | 2022 |
| CPV33 | Ayacucho | La Mar | Anco | 663537.673 | 8550219.951 | 2022 |
| CPV34 | Ayacucho | La Mar | Anco | 663436.993 | 8550119.772 | 2022 |
| CPV52 (CPV24_P2) | Ayacucho | La Mar | Chungui | 667530.41 | 8550015.74 | 2022 |
| CPV65 | Ayacucho | La Mar | Samugari | 649472.499 | 8579085.991 | 2022 |
| CPV67 | Ayacucho | La Mar | Ayna | 629366.801 | 8601537.867 | 2022 |
| CPV68 | Ayacucho | La Mar | Ayna | 629326.544 | 8601546.456 | 2022 |
| CPV69 | Ayacucho | La Mar | Ayna | 629350.797 | 8601553.535 | 2022 |
| CPV70 | Ayacucho | La Mar | Ayna | 629352.917 | 8601565.914 | 2022 |
| CPV71 | Ayacucho | La Mar | Ayna | 629353.518 | 8601578.52 | 2022 |
| CPV72 | Ayacucho | La Mar | Ayna | 629354.384 | 8601601.854 | 2022 |
| CPV73 | Ayacucho | La Mar | Ayna | 629690.381 | 8601612.047 | 2022 |
| CPV74 | Ayacucho | La Mar | Ayna | 629737.462 | 8600957.146 | 2022 |
| CPV75 | Ayacucho | La Mar | Ayna | 629730.011 | 8601608.88 | 2022 |
| CPV76 | Ayacucho | La Mar | Ayna | 628606.529 | 8602770.825 | 2022 |
| CPV77 | Ayacucho | La Mar | Ayna | 629034.848 | 8602083.121 | 2022 |
| VRAE06 | Ayacucho | La Mar | Anco | 658583 | 8567055 | 1999 |
| VRAE12 | Ayacucho | La Mar | Samugari | 641341 | 8590045 | 1999 |
| VRAE13 (VRAE15) | Ayacucho | La Mar | Santa Rosa | 642567 | 8596344 | 1999 |
| VRAE20 | Ayacucho | La Mar | Santa Rosa | 622739 | 8612954 | 1999 |
| VRAE30 | Ayacucho | La Mar | Santa Rosa | 639109 | 8599762 | 1999 |
| VRAE49 | Ayacucho | La Mar | Ayna | 627319 | 8608109 | 1999 |
| VRAE91 | Ayacucho | La Mar | Samugari | 645672 | 8587701 | 1999 |
| INDES046 | Cajamarca | Jaén | Yanuyacu | 774656 | 9371993 | 2018 |
| CER02 | Cusco | La Convención | Kimbiri | 639829 | 8600363 | 2022 |
| CHMM01_P2 | Cusco | La Convención | Kimbiri | 630770 | 8605139 | 2018 |
| CHMM07 | Cusco | La Convención | Echarati | 764952 | 8591949 | 2018 |
| CHMM11 | Cusco | La Convención | Echarati | 764762 | 8591457 | 2018 |
| CHMM12 | Cusco | La Convención | Villa Virgen | 662260 | 8561570 | 2018 |
| CPV01_P1 | Cusco | La Convención | Villa Virgen | 662246.33 | 8561568.77 | 2021 |
| CPV01_P2 | Cusco | La Convención | Villa Virgen | 662248.11 | 8561539.88 | 2021 |
| CPV01_P3 | Cusco | La Convención | Villa Virgen | 662264.48 | 8561575.41 | 2021 |
| CPV01_P4 | Cusco | La Convención | Villa Virgen | 662335.64 | 8561648.22 | 2021 |
| CPV01_P5 | Cusco | La Convención | Villa Virgen | 662343.19 | 8561660.24 | 2021 |
| CPV50 | Cusco | La Convención | Kimbiri | 639891.123 | 8600324.59 | 2022 |
| CPV78 | Cusco | La Convención | Pichari | 625895.951 | 8620248.293 | 2022 |
| CPV79 | Cusco | La Convención | Pichari | 625900.155 | 8620285.117 | 2022 |
| CPV80 | Cusco | La Convención | Pichari | 625886.94 | 8620297.839 | 2022 |
| CPV81 | Cusco | La Convención | Pichari | 625844.652 | 8620243.44 | 2022 |
| CPV82 | Cusco | La Convención | Villa Virgen | 662565.147 | 8561864.912 | 2022 |
| CPV84 | Cusco | La Convención | Villa Virgen | 668231.908 | 8553042.94 | 2022 |
| CPV85 | Cusco | La Convención | Villa Virgen | 668206.649 | 8553044.423 | 2022 |
| CPV87 | Cusco | La Convención | Villa Virgen | 661917.823 | 8561838.738 | 2022 |
| DBMC001 | Cusco | La Convención | Echarati | 742456 | 8601195 | 2022 |
| DBMC002 | Cusco | La Convención | Echarati | 742344 | 8601269 | 2022 |
| DBMC003 | Cusco | La Convención | Echarati | 742336 | 8601221 | 2022 |
| DBMC004 | Cusco | La Convención | Echarati | 764302 | 8591251 | 2022 |
| DBMC005 | Cusco | La Convención | Echarati | 764289 | 8591264 | 2022 |
| DBMC006 | Cusco | La Convención | Echarati | 764330 | 8591257 | 2022 |
| DBMC007 | Cusco | La Convención | Echarati | 765659 | 8591882 | 2022 |
| DBMC011 | Cusco | La Convención | Echarati | 763016 | 8589369 | 2022 |
| DBMC012 | Cusco | La Convención | Echarati | 763013 | 8589369 | 2022 |
| DBMC013 | Cusco | La Convención | Echarati | 763012 | 8589377 | 2022 |
| DBMC014 | Cusco | La Convención | Echarati | 763009 | 8589370 | 2022 |
| DBMC015 | Cusco | La Convención | Echarati | 763002 | 8589378 | 2022 |
| DBMC017 | Cusco | La Convención | Echarati | 769180 | 8596003 | 2022 |
| DBMC018 | Cusco | La Convención | Echarati | 769180 | 8596003 | 2022 |
| DBMC019 | Cusco | La Convención | Echarati | 769180 | 8596003 | 2022 |
| DBMC020 | Cusco | La Convención | Echarati | 769180 | 8596003 | 2022 |
| DBMC021 | Cusco | La Convención | Echarati | 769180 | 8596003 | 2022 |
| DBMC026 | Cusco | La Convención | Echarati | 769180 | 8596003 | 2022 |
| DBMC027 | Cusco | La Convención | Echarati | 769180 | 8596003 | 2022 |
| DBMC028 | Cusco | La Convención | Santa Ana | 752907 | 8586149 | 2022 |
| DBMC029 | Cusco | La Convención | Santa Ana | 752987 | 8586181 | 2022 |
| DBMC030 | Cusco | La Convención | Echarati | 763024 | 8589368 | 2022 |
| DBMC031 | Cusco | La Convención | Echarati | 763027 | 8589374 | 2022 |
| DBMC034 | Cusco | La Convención | Echarati | 762776 | 8589231 | 2022 |
| DBMC036 | Cusco | La Convención | Echarati | 769180 | 8596003 | 2022 |
| DBMC037 | Cusco | La Convención | Echarati | 769180 | 8596003 | 2022 |
| DBMC038 | Cusco | La Convención | Santa Ana | 743459 | 8573178 | 2022 |
| DBMC039 | Cusco | La Convención | Santa Ana | 743464 | 8573172 | 2022 |
| DBMC040 | Cusco | La Convención | Santa Ana | 743459 | 8573174 | 2022 |
| DBMC041 | Cusco | La Convención | Santa Ana | 743421 | 8573148 | 2022 |
| DBMC042 | Cusco | La Convención | Santa Ana | 743464 | 8573178 | 2022 |
| DBMC043 | Cusco | La Convención | Echarati | 764320 | 8591227 | 2022 |
| DBMC046 | Cusco | La Convención | Echarati | 769180 | 8596003 | 2022 |
| DBMC049 | Cusco | La Convención | Quellouno | 770482 | 8605599 | 2022 |
| DBMC050 | Cusco | La Convención | Quellouno | 770488 | 8605593 | 2022 |
| DBMC052 | Cusco | La Convención | Quellouno | 770498 | 8605591 | 2022 |
| DBMC053 | Cusco | La Convención | Quellouno | 770488 | 8605587 | 2022 |
| DBMC056 | Cusco | La Convención | Quellouno | 770503 | 8605670 | 2022 |
| DBMC057 | Cusco | La Convención | Quellouno | 770494 | 8605746 | 2022 |
| DBMC059 | Cusco | La Convención | Quellouno | 770489 | 8605724 | 2022 |
| DBMC061 | Cusco | La Convención | Quellouno | 770498 | 8605717 | 2022 |
| DBMC062 | Cusco | La Convención | Quellouno | 782326 | 8603528 | 2022 |
| DBMC063 | Cusco | La Convención | Quellouno | 782364 | 8603574 | 2022 |
| DBMC064 | Cusco | La Convención | Quellouno | 782454 | 8603560 | 2022 |
| DBMC065 | Cusco | La Convención | Quellouno | 782455 | 8603563 | 2022 |
| DBMC066 | Cusco | La Convención | Quellouno | 782332 | 8603528 | 2022 |
| DBMC067 | Cusco | La Convención | Quellouno | 782345 | 8603529 | 2022 |
| DBMC068 | Cusco | La Convención | Quellouno | 782376 | 8603548 | 2022 |
| DBMC069 | Cusco | La Convención | Quellouno | 782444 | 8603542 | 2022 |
| DBMC070 | Cusco | La Convención | Quellouno | 782436 | 8603565 | 2022 |
| DBMC071 | Cusco | La Convención | Quellouno | 782422 | 8603556 | 2022 |
| DBMC072 | Cusco | La Convención | Quellouno | 782414 | 8603553 | 2022 |
| DBMC073 | Cusco | La Convención | Quellouno | 782406 | 8603577 | 2022 |
| DBMC074 | Cusco | La Convención | Quellouno | 782462 | 8603543 | 2022 |
| DBMC075 | Cusco | La Convención | Quellouno | 782586 | 8603546 | 2022 |
| DBMC076 | Cusco | La Convención | Quellouno | 782599 | 8603557 | 2022 |
| DBMC077 | Cusco | La Convención | Quellouno | 782614 | 8603561 | 2022 |
| DBMC078 | Cusco | La Convención | Quellouno | 782617 | 8603561 | 2022 |
| DBMC079 | Cusco | La Convención | Quellouno | 782642 | 8603567 | 2022 |
| DBMC080 | Cusco | La Convención | Quellouno | 782648 | 8603564 | 2022 |
| DBMC081 | Cusco | La Convención | Echarati | 782618 | 8603544 | 2022 |
| DBMC082 | Cusco | La Convención | Echarati | 745924 | 8606147 | 2022 |
| DBMC083 | Cusco | La Convención | Echarati | 745924 | 8606147 | 2022 |
| DBMC084 | Cusco | La Convención | Echarati | 745924 | 8606147 | 2022 |
| DBMC085 | Cusco | La Convención | Echarati | 729141 | 8602143 | 2022 |
| DBMC086 | Cusco | La Convención | Echarati | 729137 | 8602091 | 2022 |
| DBMC087 | Cusco | La Convención | Echarati | 729137 | 8602091 | 2022 |
| DBMC088 | Cusco | La Convención | Echarati | 729268 | 8602143 | 2022 |
| DBMC089 | Cusco | La Convención | Quellouno | 729263 | 8602160 | 2022 |
| DBMC090 | Cusco | La Convención | Quellouno | 761569 | 8600899 | 2022 |
| DBMC091 | Cusco | La Convención | Quellouno | 761573 | 8600928 | 2022 |
| DBMC092 | Cusco | La Convención | Quellouno | 761587 | 8600908 | 2022 |
| DBMC093 | Cusco | La Convención | Quellouno | 761608 | 8600917 | 2022 |
| DBMC094 | Cusco | La Convención | Quellouno | 761612 | 8600909 | 2022 |
| DBMC095 | Cusco | La Convención | Quellouno | 761508 | 8600973 | 2022 |
| DBMC096 | Cusco | La Convención | Quellouno | 761640 | 8600810 | 2022 |
| DBMC097 | Cusco | La Convención | Quellouno | 761640 | 8600810 | 2022 |
| DBMC098 | Cusco | La Convención | Quellouno | 761718 | 8600760 | 2022 |
| DBMC099 | Cusco | La Convención | Quellouno | 761718 | 8600760 | 2022 |
| DBMC100 | Cusco | La Convención | Echarati | 761706 | 8600757 | 2022 |
| DBMC101 | Cusco | La Convención | Santa Ana | 763299 | 8587506 | 2022 |
| DBMC102 | Cusco | La Convención | Santa Ana | 752433.14 | 8584692.27 | 2022 |
| DBMC103 | Cusco | La Convención | Santa Ana | 753542.6 | 8584313.1 | 2022 |
| DBMC104 | Cusco | La Convención | Santa Ana | 753523.10 | 8584292.51 | 2022 |
| DBMC105 | Cusco | La Convención | Santa Ana | 753513.66 | 8584279.32 | 2022 |
| DBMC106 | Cusco | La Convención | Santa Ana | 753552.85 | 8584228.64 | 2022 |
| DBMC107 | Cusco | La Convención | Santa Ana | 753549.51 | 8584223.37 | 2022 |
| DBMC108 | Cusco | La Convención | Santa Ana | 753549.23 | 8584214.30 | 2022 |
| VILLA_VISTA | Cusco | La Convención | Unión Ashaninka | 612784 | 8634821 | 1999 |
| VRAE02 | Cusco | La Convención | Villa Virgen | 661539 | 8563443 | 1999 |
| VRAE99 | Cusco | La Convención | Pichari | 624747 | 8624564 | 1999 |
| INIA_PM01 | Madre de Dios | Inambari | Santa Rosa | 361204 | 8571949 | 2022 |
| INIA_PM02 | Madre de Dios | Inambari | Santa Rosa | 361207 | 8571950 | 2022 |
| INIA_PM03 | Madre de Dios | Inambari | Santa Rosa | 356559 | 8571184 | 2022 |
| INIA_PM04 | Madre de Dios | Inambari | Santa Rosa | 358631 | 8571774 | 2022 |
| INIA_PM05 | Madre de Dios | Inambari | C.N. Arazaire | 353702 | 8556455 | 2022 |
| INIA_PM06 | Madre de Dios | Inambari | C.N. Arazaire | 354154 | 8562124 | 2022 |
| INIA_PM07 | Madre de Dios | Inambari | Santa Rosa | 356555 | 8571501 | 2022 |
| INIA_PM08 | Madre de Dios | Inambari | Puerto Trujillo | 357086 | 8574776 | 2022 |
| INIA_PM09 | Madre de Dios | Inambari | Puerto Trujillo | 357087 | 8574750 | 2022 |
| INIA_PM10 | Madre de Dios | Inambari | Puerto Trujillo | 357096 | 8574761 | 2022 |
| DBMC_A1 | Piura | Huncabamba | Huarmaca | 53143.8 | 794037.5 | 2022 |
| DBMC_A2 | Piura | Huncabamba | Huarmaca | 53143.3 | 794028.2 | 2022 |
| DBMC_A3 | Piura | Huncabamba | Huarmaca | 53142.4 | 794029 | 2022 |
| DBMC_A4 | Piura | Huncabamba | Huarmaca | 53140.2 | 794023.2 | 2022 |
| DBMC_A5 | Piura | Huncabamba | Huarmaca | 53141.3 | 794018.2 | 2022 |
| DBMC_A6 | Piura | Huncabamba | Huarmaca | 53140.2 | 794019.1 | 2022 |
| DBMC_A7 | Piura | Huncabamba | Huarmaca | 53148.9 | 794049.6 | 2022 |
| DBMC_A8 | Piura | Huncabamba | Huarmaca | 53150.2 | 794051.6 | 2022 |
| DBMC_A10 | Piura | Huncabamba | Huarmaca | 53147.7 | 794101.9 | 2022 |
| DBMC_A11 | Piura | Huncabamba | Huarmaca | 53143.8 | 794037.5 | 2022 |
| DBMC_A12 | Piura | Huncabamba | Huarmaca | 53143.8 | 794037.5 | 2022 |
| DBMC_A13 | Piura | Huncabamba | Huarmaca | 53115 | 794115.1 | 2022 |
| DBMC_A14 | Piura | Huncabamba | Huarmaca | 53140.2 | 794019.1 | 2022 |
| DBMC_A15 | Piura | Huncabamba | Huarmaca | 53115 | 794115.1 | 2022 |
| DBMC_A16 | Piura | Huncabamba | Huarmaca | 53115 | 794115.1 | 2022 |
| DBMC_A17 | Piura | Huncabamba | Huarmaca | 53140.2 | 794023.2 | 2022 |
| DBMC_A18 | Piura | Huncabamba | Huarmaca | 53106.4 | 794122.7 | 2022 |
| DBMC_A19 | Piura | Huncabamba | Huarmaca | 53143.8 | 794037.5 | 2022 |
| DBMC_A20 | Piura | Huncabamba | Huarmaca | 53148.9 | 794049.6 | 2022 |
| DBMC_P1 | Piura | Huncabamba | Canchaque | 52125.1 | 793618.3 | 2022 |
| DBMC_P2 | Piura | Huncabamba | Canchaque | 52128.2 | 793619.4 | 2022 |
| DBMC_P3 | Piura | Huncabamba | Canchaque | 52129.9 | 793622.2 | 2022 |
| DBMC_P4 | Piura | Huncabamba | Canchaque | 52127.2 | 793617.8 | 2022 |
| DBMC_P5 | Piura | Huncabamba | Canchaque | 52128.6 | 793607.8 | 2022 |
| DBMC_P6 | Piura | Huncabamba | Canchaque | 52128.7 | 793557.7 | 2022 |
| DBMC_P7 | Piura | Huncabamba | Canchaque | 52128.5 | 793546.7 | 2022 |
| DBMC_P8 | Piura | Huncabamba | Canchaque | 52126.5 | 793541.8 | 2022 |
| DBMC_P9 | Piura | Huncabamba | Canchaque | 52126.2 | 793537.7 | 2022 |
| DBMC_P10 | Piura | Huncabamba | Canchaque | 52125.4 | 793542.6 | 2022 |
| DBMC_P11 | Piura | Huncabamba | Canchaque | 52054.1 | 793553.6 | 2022 |
| DBMC_P12 | Piura | Huncabamba | Canchaque | 52050.9 | 793543.6 | 2022 |
| DBMC_P13 | Piura | Huncabamba | Canchaque | 52041.8 | 793544 | 2022 |
| DBMC_P14 | Piura | Huncabamba | Canchaque | 52042.7 | 793551.5 | 2022 |
| DBMC_P15 | Piura | Huncabamba | Canchaque | 52128.6 | 793607.8 | 2022 |
| DBMC_P16 | Piura | Huncabamba | Canchaque | 52128.2 | 793619.4 | 2022 |
| DBMC_P17 | Piura | Huncabamba | Canchaque | 52128.6 | 793607.8 | 2022 |
| DBMC_P18 | Piura | Huncabamba | Canchaque | 52054.1 | 793553.6 | 2022 |
| DBMC_P19 | Piura | Huncabamba | Canchaque | 52128.2 | 793619.4 | 2022 |
| DBMC_P20 | Piura | Huncabamba | Canchaque | 52128.2 | 793619.4 | 2022 |
| DBMC_R1 | Piura | Huncabamba | Huarmaca | 53129.8 | 793931.4 | 2022 |
| DBMC_R2 | Piura | Huncabamba | Huarmaca | 53133.4 | 793932.4 | 2022 |
| DBMC_R3 | Piura | Huncabamba | Huarmaca | 53140.1 | 793933.7 | 2022 |
| DBMC_R4 | Piura | Huncabamba | Huarmaca | 53148.8 | 793938.5 | 2022 |
| DBMC_R5 | Piura | Huncabamba | Huarmaca | 53143.7 | 794010.1 | 2022 |
| DBMC_R6 | Piura | Huncabamba | Huarmaca | 53143.4 | 794027.9 | 2022 |
| DBMC_R7 | Piura | Huncabamba | Huarmaca | 53143.6 | 794037 | 2022 |
| DBMC_R8 | Piura | Huncabamba | Huarmaca | 53149.1 | 794049.4 | 2022 |
| DBMC_R9 | Piura | Huncabamba | Huarmaca | 53149.8 | 794051.2 | 2022 |
| DBMC_R10 | Piura | Huncabamba | Huarmaca | 53147.7 | 794101.9 | 2022 |
| DBMC_R11 | Piura | Huncabamba | Huarmaca | 53143.7 | 794010.1 | 2022 |
| DBMC_R12 | Piura | Huncabamba | Huarmaca | 53140.2 | 794023.2 | 2022 |
| DBMC_R13 | Piura | Huncabamba | Huarmaca | 53141.3 | 794018.2 | 2022 |
| DBMC_R14 | Piura | Huncabamba | Huarmaca | 53140.2 | 794019.1 | 2022 |
| DBMC_R15 | Piura | Huncabamba | Huarmaca | 53148.9 | 794049.6 | 2022 |
| DBMC_R16 | Piura | Huncabamba | Huarmaca | 53150.2 | 794051.6 | 2022 |
| DBMC_R17 | Piura | Huncabamba | Huarmaca | 53147.7 | 794101.9 | 2022 |
| DBMC_R18 | Piura | Huncabamba | Huarmaca | 53148.9 | 794049.6 | 2022 |
| DBMC_R19 | Piura | Huncabamba | Huarmaca | 53148.9 | 794049.6 | 2022 |
| DBMC_R20 | Piura | Huncabamba | Huarmaca | 53147.7 | 794101.9 | 2022 |
| INDES101 | San Martín | Mariscal Cáceres | Llanos | 280716 | 9203411 | 2018 |
| INDES106 | San Martín | Mariscal Cáceres | Soledad | 766769 | 9186654 | 2018 |
| INDES112 | San Martín | Mariscal Cáceres | Soledad | 559050 | 1412541 | 2018 |
| CMM02 | Ucayali | Atalaya | Raymondi | 640369 | 8812641 | 2018 |
