# Supplementary material for: Genetic structure of traditional cacao reveals four new genetic lineages in indigenous Amazonian sites in Peru
Source: PLoS One. 2026 Jul 6;21(7):e0351690. doi: 10.1371/journal.pone.0351690 (PMC13336180; doi:10.1371/journal.pone.0351690)
Supplement: S3 Table — Phase III dataset has the 10 simulated populations of Motamayor et al. (2008), members from four phylogenetic clades, five Amelonado-Nacional accessions, CCN 51 and five Amelonado-Criollo accessions. (DOCX) [file pone.0351690.s003.docx]

**Genetic structure of traditional cacao reveals four new genetic lineages in indigenous Amazonian sites in Peru**

**Supplemental Table S3.** Summary details of iterations at different cluster assignment with Phase III dataset. Phase III dataset has the 10 simulated populations of Motamayor et al. (2008), members from four phylogenetic clades, five Amelonado-Nacional accessions, CCN 51 and five Amelonado-Criollo accessions

| **K (number runs)** | **Clade I**  **(22 individuals)** | **Clade II**  **(24 individuals)** | **Clade III**  **(63 individuals)** | **Clade IV**  **(97 individuals)** | **CCN 51**  **(1 individual)** |
| --- | --- | --- | --- | --- | --- |
| 11  (10 runs) | mainly admixed with Clade IV (all runs) in variable combination with Iquitos, Nanay, Amelonado, Criollo. One run with 9 pure members in new group. | mainly admixed with Clade III (9 runs) in variable combination with Iquitos, Amelonado, Criollo. One run with Clade II as new group. | New group (2 runs) or equivalent to Nacional (8 runs) | Mainly new group (7 runs) or equivalent to Contamana (3 runs) | Mainly admixed as Amelonado/Iquitos/Criollo (9 runs). One run as Clade II mixed with Amelonado, Iquitos, Criollo, Nanay |
| 12 (10 runs) | mainly admixed as Clade I/ Clade IV/other (4 runs), Clade II/Clade IV/other (4 runs), Clade IV/other (2 runs). Other are variable combinations of all 10 reference groups except Guiana. Four runs with 9 pure members in new group. | Four runs with all but 2-3 members of Clade II in new group.  Mainly admixed as Nacional/other (6 runs) or Clade III/other (2 runs) with other as variable contributions from Iquitos, Amelonado, Criollo, Marañon. | Two runs with Clade III as new group.  Mainly allocated as  Nacional (8 runs). Some individuals admixed as Nacional/other (4 runs) with other as variable contribution from Clade I, Clade II, Curaray, Marañon. | New group (all members, all 10 runs) | Mainly admixed as Amelonado/Iquitos/Criollo (6 runs). Four runs as Clade II mixed with other. Other always include Amelonado, Iquitos, Criollo. One run with Nanay contribution. |
| 13 (15 runs) | 9 pure members (11 of 15 runs) or admixed (majority or all) individuals with contributions from Nanay & Clade IV (all runs) with frequent contributions from Iquitos & Marañon (13-14 runs) and variable contributions from Clades I, II, III, Amelonado, Contamana, Purus and CCN 51. | New group (6 of 15 runs) or all admixed (7 of 15 runs). Admixed with Clade III (9 of 15 runs). Admixed individuals present as Clade II/Clade III (3 of 15 runs); Clade III, Iquitos, Criollo with variable other groups (4 of 15 runs); Clade II/Nacional (6 of 15 runs); and Clade III/CCN 51 (2 runs) | New group (all individuals in 10 of 15 runs) or Nacional with variable contribution from Clade III (5 of 15 runs). | New group (all individuals in 14 of 15 runs) or equivalent to Contamana (1 of 15 runs). | New group as only pure member (4 of 15 runs). Adnixed with major contribution from Clade II, Amelonado, Criollo, Iquitos & Nanay (6 of 15 runs) or admixed as Iquitos, Amelonado and Criollo (5 of 15 runs) . |
| 14  (10 runs) | pure and admixed with Clade IV (all runs) | all admixed as Clade II/Clade III (7 runs) or majority pure (3 runs). | new group (all members, all 10 runs) | new group (all members, all 10 runs) | either ~99% Clade II (7 runs) or admixed as 72-87% Clade II with additional from 3 – 4 groups (Amelonado, Nanay, Iquitos, Criollo). |
