# Supplementary material for: Genetic structure of traditional cacao reveals four new genetic lineages in indigenous Amazonian sites in Peru
Source: PLoS One. 2026 Jul 6;21(7):e0351690. doi: 10.1371/journal.pone.0351690 (PMC13336180; doi:10.1371/journal.pone.0351690)
Supplement: S1 Fig — (DOCX) [file pone.0351690.s004.docx]

**Genetic structure of traditional cacao reveals four new genetic lineages in indigenous Amazonian sites in Peru**


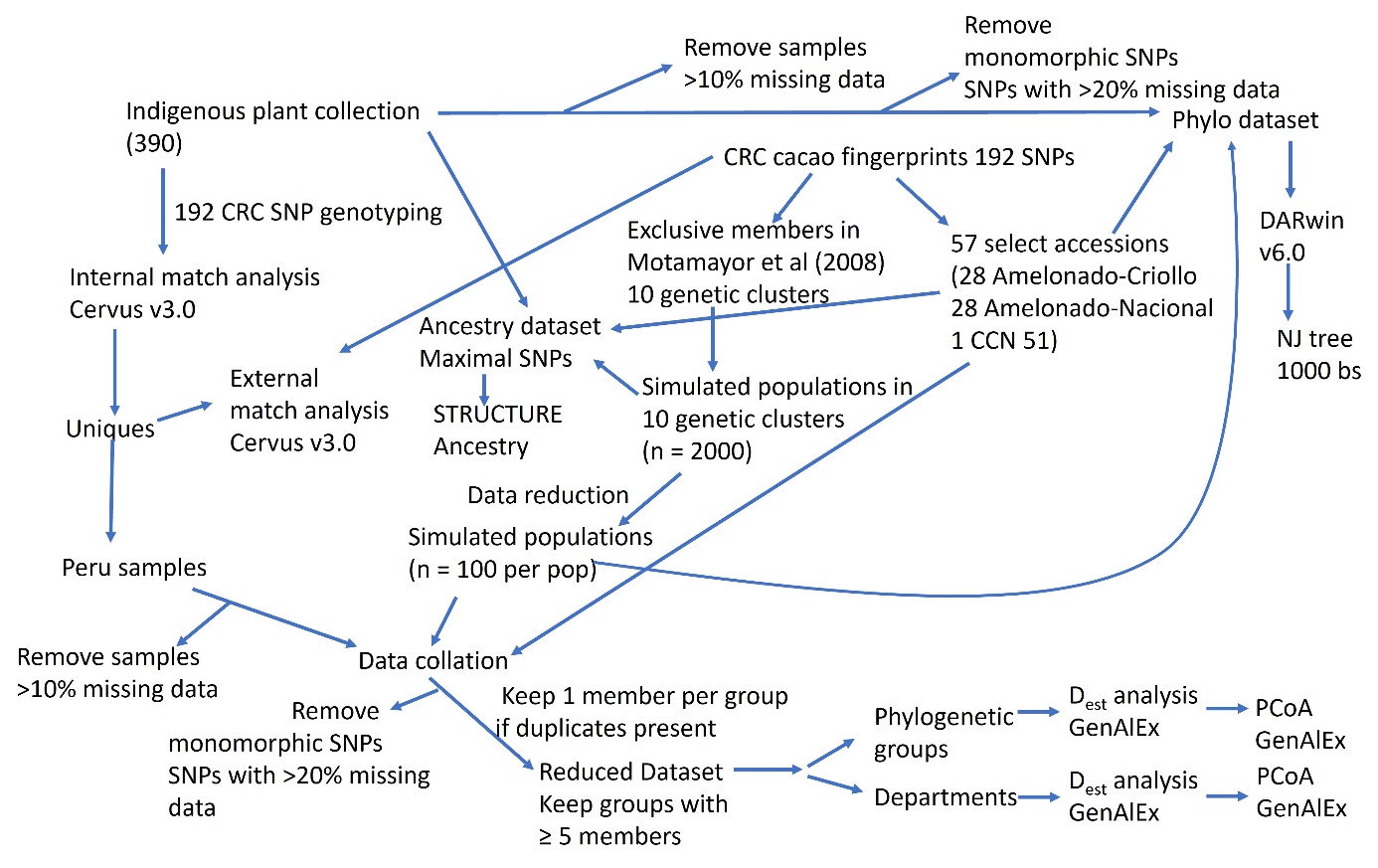


**Supplemental Figure 1.** Workflow summarising graphically the methodology.
