# Supplementary material for: Genetic structure of traditional cacao reveals four new genetic lineages in indigenous Amazonian sites in Peru
Source: PLoS One. 2026 Jul 6;21(7):e0351690. doi: 10.1371/journal.pone.0351690 (PMC13336180; doi:10.1371/journal.pone.0351690)
Supplement: S2 Fig — (DOCX) [file pone.0351690.s005.docx]

**Genetic structure of traditional cacao reveals four new genetic lineages in indigenous Amazonian sites in Peru**


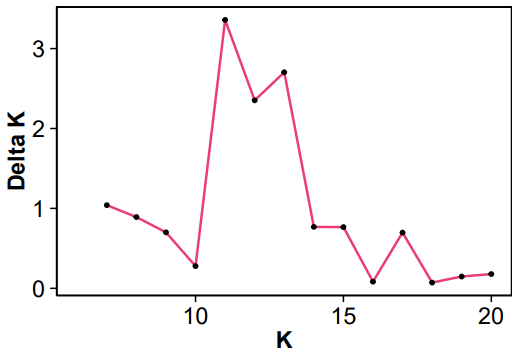


**Supplemental Figure 2**. Preliminary Evanno plot of samples collected in Peru (390) with 10 simulated populations.
