# Supplementary material for: Genetic structure of traditional cacao reveals four new genetic lineages in indigenous Amazonian sites in Peru
Source: PLoS One. 2026 Jul 6;21(7):e0351690. doi: 10.1371/journal.pone.0351690 (PMC13336180; doi:10.1371/journal.pone.0351690)
Supplement: S3 Fig — (DOCX) [file pone.0351690.s006.docx]

**Genetic structure of traditional cacao reveals four new genetic lineages in indigenous Amazonian sites in Peru**


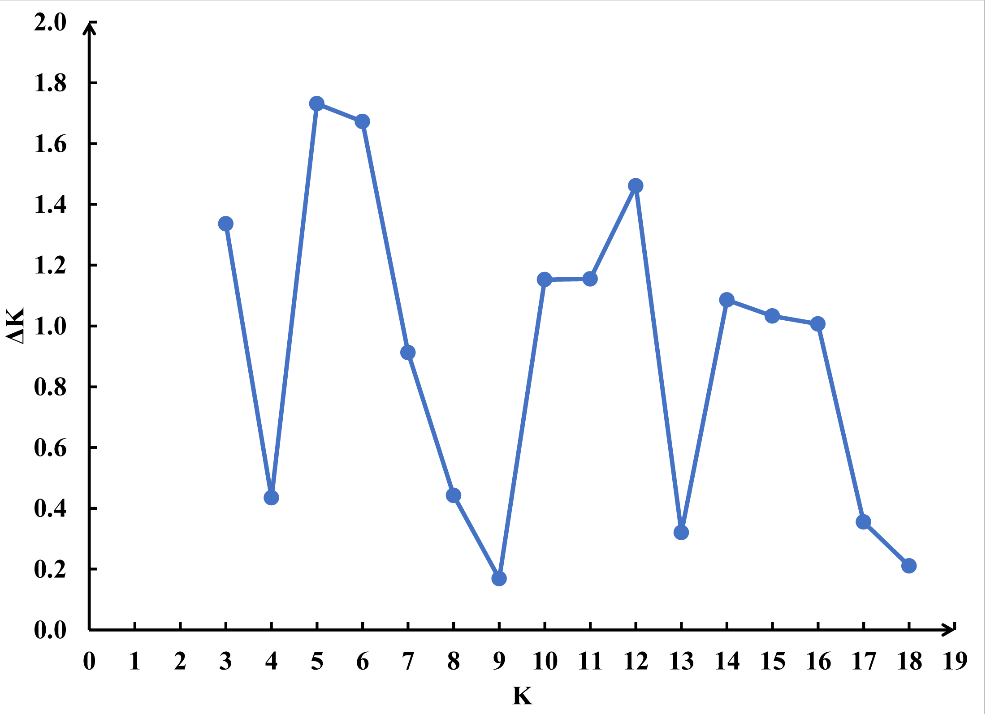


**Supplemental Figure 3**. Evanno plot based on dataset of simulated population clusters of Motamayor et al. [8] and pure group members in four phylogenetic clades.
